# A Uniquely Altered Oral Microbiome Composition Was Observed in Pregnant Rats With *Porphyromonas gingivalis* Induced Periodontal Disease

**DOI:** 10.3389/fcimb.2020.00092

**Published:** 2020-03-06

**Authors:** Molly S. Walkenhorst, Leticia Reyes, Gonzalo Perez, Ann Progulske-Fox, Mary B. Brown, Priscilla L. Phillips

**Affiliations:** ^1^Department of Microbiology and Immunology, Kirksville College of Osteopathic Medicine, A.T. Still University of Health Sciences, Kirksville, MO, United States; ^2^Department of Pathobiological Sciences, School of Veterinary Medicine, University of Wisconsin-Madison, Madison, WI, United States; ^3^Department of Oral Biology, College of Dentistry, University of Florida, Gainesville, FL, United States; ^4^Department of Infectious Diseases and Immunology, College of Veterinary Medicine, University of Florida, Gainesville, FL, United States

**Keywords:** oral microbiome, oxygen requirement, microbial metabolites, pregnancy, periodontal disease, rat model

## Abstract

*Porphyromonas gingivalis* is an anaerobic bacterium commonly found in the oral cavity and associated with the development of periodontal disease. *P. gingivalis* has also been linked to several systemic vascular and inflammatory diseases including poor pregnancy outcomes. Little is known about the changes in the oral flora during pregnancy in connection to *P. gingivalis* infection. This pilot study aims to explore changes in the oral microbiome due to *P. gingivalis* inoculation and pregnancy in an *in vivo* rat model of periodontal disease. A metagenomic sequencing analysis targeting seven of the 16S rRNA gene variable regions was performed for oral samples collected at the following time points: baseline control (week 0), *P. gingivalis* inoculated (week 11), *P. gingivalis* inoculated and pregnant rat at necropsy (week 16). A second set of animals were also sampled to generate a sham-inoculated (week 11) control group. We found that the rat oral microbiome profiles were more similar to that of the human oral cavity compared to previous reports targeting one or two 16S variable regions. Overall, there appears to be a relatively stable core microbiome in the oral cavity. As expected, *P. gingivalis* induced periodontal disease resulted in oral microbiome dysbiosis. During pregnancy, some aspects of the oral microbiome shifted toward a more baseline-like profile. However, population analyses in terms of dissimilarity measures and especially metagenomic based predictions of select characteristics such as cell morphology, oxygen requirement, and major metabolite synthesis showed that pregnancy did not restore the composition of the oral microbiome. Rather, a uniquely altered oral microbiome composition was observed in pregnant rats with pre-established periodontal disease.

## Introduction

Periodontitis is currently considered a disease of dysbiotic polymicrobial etiology in which oral microbial species capable of thriving in inflamed microenvironments predominate (Abusleme et al., 2013; Hajishengallis, 2015). Although the pathogenesis of periodontal disease is complex, one view is that dysbiosis is initiated by the presence of keystone species that subverts the host immune defense, and this allows secondary pathogens to thrive and ultimately dominate. *Porphyromonas gingivalis* has gained notoriety as a keystone pathogen of periodontal disease because even as a low abundance species, it has profound effects on the oral microbial community structure (Hajishengallis, 2011; Olsen et al., 2017). *P. gingivalis* is armed with an array of virulence factors that subvert host innate antimicrobial defenses without blocking inflammatory pathways; referred to as “non-productive inflammation” (Zenobia and Hajishengallis, 2015). This not only supplies *P. gingivalis* the nutrient source it needs for growth, but also provides a selective advantage for co-colonizing microbes able to tolerate and thrive in an inflammatory environment (Zenobia and Hajishengallis, 2015).

Metagenomic studies of the oral microbiome also show that pregnancy perturbs the composition of the oral microbial community (Paropkari et al., 2016; Lin et al., 2018). In pregnant women without periodontal disease, the supragingival community structure is reported to shift toward a predominance of bacteria within the genera *Neisseria, Porphyromonas*, and *Treponema* (Lin et al., 2018). In particular, the increased abundance of *Prevotella* and *Treponema* spp. is linked to increasing concentrations of sex hormones associated with pregnancy (Lin et al., 2018). Moreover, pregnancy can modify the impact of environmental risk factors on the oral microbiome. For instance, the subgingival microbiome of women who smoke during pregnancy are reported to have significantly less Gram-negative and Gram-positive anaerobes than non-pregnant smokers (Paropkari et al., 2016). To date, it is unknown if or in what manner pregnancy affects the dysbiotic oral microbiome associated with periodontal disease in patients with preexisting oral disease.

For this pilot study, we chose *P. gingivalis* as our model organism since it has been implicated in promoting adverse pregnancy outcomes (Barak et al., 2007; Chaparro et al., 2013; Vanterpool et al., 2016). Our objective was to investigate the interaction of pregnancy and the oral dysbiosis of periodontal disease in a rat model of *P. gingivalis*-induced periodontitis (Kesavalu et al., 2007; Verma et al., 2010) using 16S metagenomic analysis.

## Materials and Methods

### Periodontitis Model and Sample Collection

All procedures were conducted after approval from the University of Wisconsin–Madison, Institutional Animal Care and Use Committee. Specific pathogen free CD-IGS rats obtained from the same colony (Charles River International Laboratories, Inc., Kingston, NY; RGD ID 734476) were housed in the same room under barrier conditions. Oral swabs and blood were collected prior to antibiotic treatment and animals were randomly assigned to sham control (inoculation with sterile vehicle) or *P. gingivalis* strain A7UF, a lab adapted strain expressing Type IV *fim* operon and with 99.2% whole genome DNA sequence identity to A7436 (data not shown). At the onset of the inoculation phase of the study and thereafter control animals were always handled before infected animals.

Periodontitis was induced as previously described (Phillips et al., 2018). Specifically, 7–8 week old female rats first received kanamycin (20 mg) and ampicillin (20 mg) daily for 4 days in the drinking water to reduce the number of commensal bacteria. The oral cavity was then swabbed with 0.12% chlorhexidine gluconate (Peridex: 3M ESPE Dental Products, St. Paul, MN, USA) mouth rinse to inhibit endogenous organisms and to facilitate subsequent colonization with *P. gingivalis*. Rats were switched back to antibiotic free water and rested for 3 days to allow clearance of antibiotics from their system before beginning the inoculation phase of the study. An equal number of rats were randomly assigned to the control or *P. gingivalis* group. Each inoculate was prepared from an overnight culture of *P. gingivalis* grown in supplemented Tryptic Soy Broth (Phillips et al., 2018). Bacterial concentrations in broth were determined by optical density readings taken at 550 nm with a UV-6300 PC double beam spectrophotometer (VWR, Chicago, IL, USA). Bacteria were pelleted by centrifugation at 3,000 × g for 5 min and resuspended in sterile 2% (w/v) low viscosity carboxymethylcellulose (CMC; Sigma, St. Louis, MO, USA) to achieve a final concentration of 1 × 10^10^ CFU per ml. Each animal received an oral inoculation containing 1 × 10^9^ CFU for 4 consecutive days per week on 6 alternate weeks totaling 24 inoculations over a 12-week period. Control animals received sterile 2% CMC. In order to minimize disruption of bacterial plaque, animals were fed a gamma-irradiated powdered rodent diet (Teklad Global 18% protein rodent diet, Envigo, Madison, WI) during the inoculation phase of the study. At the end of the inoculation period (week 12), oral swabs were taken, blood was collected for serology, and rats were switched back to the same diet in a pelleted form. Animals were rested for 1 week before breeding. Breeding was confirmed by the presence of sperm in vaginal lavages that were performed the following morning, which was considered gestation day GD 0. Dams were euthanized at GD 18 oral swabs were taken, skulls were collected for morphometric analysis of alveolar bone loss, and serum was collected for detection of *P. gingivalis* specific IgM and IgG.

For the purposes of 16S metagenomic analysis, we were interested in the oral samples collected from sham and *P. gingivalis* treated CD rats. Specifically, we processed oral specimens collected from the *P. gingivalis* (PG) treatment group (six animals) at three time points for sequencing: baseline, 3-months post *P. gingivalis* inoculation, and at necropsy (pregnant at GD 18). In contrast, only oral specimens collected from the sham treatment group (six animals) at the 3-months post sham inoculation time point were used for sequencing. The animals and specimen descriptions, including the sample IDs for the 24 sequencing libraries we generated and used for this 16S metagenomic analysis pilot study, are listed in Table S1.

### Assessment of Alveolar Bone Resorption

Mandibles were disarticulated from the skull and most of the tissue was removed by dissection. Remaining tissue was removed by Dermestid beetles. Rat mandibles were then immersed in a 3% (v/v) hydrogen peroxide solution overnight, washed with deionized water and air-dried. In order to delineate the cemento-enamel junction (CEJ), jaws were stained with a 0.1% (W/V) methylene blue for 1 min, washed and air-dried. Specimens were coded to prevent bias and calibrated images of both the buccal and lingual side of each jaw were captured with an EOS 650D RebelT41 camera (Canon, USA, Inc., Long Island, NY). Morphometric measurements were performed by two independent investigators that were blinded to treatment. Calibrated digital images were analyzed with Image J 1.50b analysis software (Rasband, National Institutes of Health, USA). Area measurements (mm^2^) of both the lingual and buccal surface of each mandible were determined by using the freehand tool to manually trace the surface perimeter of the CEJ and alveolar bone crest (ABC). The sum of both area measurements was recorded and values from two independent investigators were averaged.

Maxillae were fixed in 10% buffered formalin, washed and decalcified for 48 h before processing for histology. Excess tissue was removed and the row of molars were transected through the center of each molar so that interdental bone loss could be evaluated. Calibrated images of maxillary tissue were analyzed with Image J software as previously described (Phillips et al., 2018). Briefly, a line was drawn to connect the mesial to the distal aspect of each molar at the CEJ. A central line perpendicular to this CEJ was drawn from the CEJ to the top of the alveolar bone crest and the distance was recorded in mm. The distance measurements of each interdental region were averaged.

### Detection of *P. gingivalis*-Specific IgM and IgG

Humoral responses to *P. gingivalis* were determined by ELISA as previously described (Phillips et al., 2018) with the following modifications. Briefly, a whole cell lysate of *P. gingivalis* was sonicated (Sonic Dismembrator, Thermo Fisher Scientific, Waltham, MA) and the total protein concentration of the lysate was measured by Bradford Protein Assay (Thermo Fisher Scientific, Waltham, MA). Stock aliquots of the lysate were stored at a concentration of 2 mg/ml at −20 C. For the ELISA assay, whole cell lysates were diluted with 50 mM bicarbonate coating buffer pH 9.4 (Thermo Fisher Scientific, Waltham, MA) to yield a final concentration of 20 μg/ml. Goat anti-rat IgM (Southern Biotech, Birmingham, AL) and donkey anti-rat IgG (Thermo Fisher Scientific, Waltham, MA) conjugated to horse radish peroxidase (HRP) were used for detection (1:4,000 and 1:2,500, respectively). Pooled sera from *P. gingivalis* infected rats and uninfected animals were used to establish serum dilutions that fell within the linear range of a dilution curve. Subsequent ELISAs were batched and each plate contained a positive and negative control. In order to minimize plate to plate variation, each 96 well plate contained serum samples from each group. All samples were run in duplicate and readings were obtained with a Model 680 microplate reader (Biorad Laboratories, Hercules, CA).

Statistical analysis of alveolar bone loss measurements and serology data were performed with unpaired student's *t*-test using Prism 7.03 Software (GraphPad Software Inc.). For all testing *P* < 0.05 was considered significantly different.

### DNA Isolation and Enrichment for Bacterial DNA

The specimens for each sampled time point were collected on either rayon swabs (sham-inoculated) or cytological brushes (baseline, PG-inoculated, pregnant) and flash frozen and stored at −80°C until they were batch processed. At the time of processing, collection devices were thawed, immersed in sterile 200 μL of 1× phosphate buffered saline (PBS; pH 7.4), vortexed for 30 s, and the solution was transferred into sterile 2 mL sample extract tube. The swab/brush was centrifuged at 15,000 × *g* for 5 min to collect the residual liquid that was trapped in the collection device, then pipetting it into the appropriate sample extract tube. This washing process was repeated twice more for a total of three rinses for each swab. All extracted liquids from each sample were combined into one tube per specimen. Each sample extract tube was centrifuged at 21,000 × *g* for 10 min to pellet cells and detritus. The PBS was removed from the pellet and 40 μL of resuspended Dynabeads from the Dynabeads DNA DIRECT Universal Kit (Invitrogen: Cat. No. 63006) was added to each sample pellet and mixed. The Dynabead/sample mixtures were processed according to the manufacturers' protocol and the concentration of the isolated total DNA in the eluent was estimated using a NanoDrop 2000C Spectrophotometer (Thermo Scientific: Cat. No. ND2000C). The total DNA isolated from each oral specimen contains a large proportion of rat DNA, thus it is essential to enrich for bacterial DNA to optimize downstream 16S metagenomic analysis. The chemistry provided in the NEBNext Microbiome DNA Enrichment Kit (New England BioLabs: Cat. No. E2612L) enriches for bacterial DNA by selectively binding and removing CpG-methylated host DNA while maintaining microbial diversity after enrichment. Cytosine methylation occurs in bacteria in very low frequency across bacterial genomes while it is common across eukaryotic genomes. Validation studies, referenced by the manufacturer, using this enrichment method have shown that bacterial species with unusual methylation density of its DNA are rare and bind at a very low level to the enrichment beads (e.g., *Neisseria flavescens*). The enriched microbial DNA purified by the ethanol precipitation and the DNA concentration was estimated using a NanoDrop 2000c spectrophotometer. Semi-quantitative verification of enrichment was performed using qPCR (CFX-Connect Real-Time PCR detection system; BioRad Laboratories Inc.), SsoAdvanced Universal SYBR Green Supermix (BioRad Laboratories Inc.: Cat. No. 1725270), and NEBNext 16S Universal Control Primers (New England BioLabs).

### PCR Amplification of Microbial 16S rRNA Gene Variable Regions

16S rRNA libraries of each microbial DNA enriched sample were generated by PCR using the Ion 16S Metagenomics Kit (ThermoFisher Scientific: Cat. No. A26216) and a PCR thermocycler (Mastercycler nexus gradient; Eppendorf AG). Briefly, two primer sets are used to generate amplicons from six different regions of the 16S rRNA genes. Primer Set One targets variable regions 2, 4, 8 and Primer Set Two targets variable regions 3, 6–7, 9. PCR amplification was performed according to the manufacturers' protocol for each sample, along with negative and positive controls. The amplicons from 20 μL of each PCR reaction were then purified using the Axygen AxyPrep Mag PCR Clean-up Kit (Fisher Scientific: Cat. No. 14-223-151) and eluted in elution buffer (EB; 10 mM Tris-Cl, pH 8.5). The amplicons generated for each pair of reactions (two primer sets) for each sample were pooled and their DNA concentration was estimated with a NanoDrop 2000c spectrophotometer until confirmation that each sample pool consisted of 100 ng−1 μg of DNA, as required for adaptor ligation. The final concentration of amplicons in each sample pool was determined using a Qubit dsDNA hs Assay Kit (Invitrogen: Cat. No. Q32851) according to the manufacturers' protocol.

### 16S Barcoded Library Preparation for Multiplexed Sequencing

Sequencing-ready16S rRNA gene libraries were prepared using the NEBNext Fast DNA Library Prep Set for Ion Torrent (New England BioLabs: Cat. No. E6270S) and DNA barcode-adapter oligonucleotides specific for the Ion Torrent system. All 16s rRNA libraries were prepared according to the manufactures' recommendations with modifications to maximize yield. Briefly, purified 16S amplicons were first end repaired and blunt-end ligated to sample specific barcode-adapters (Table S2) in order to generate three groups for downstream pooling. We estimated that the maximum number of libraries to load in equal-molar concentrations per 318™ Ion Chip in order to generate a sufficient number of reads for metagenomic analysis was 13. Adaptor ligated DNA was size selected to obtain fragments between 200 and 400 base pairs long using the Agencourt AMPure XP PCR Purification system (Beckman Coulter: Cat. No. A63880). DNA fragments below 200 base pairs in length would likely consist of free adaptors and empty adaptor-adaptor products, whereas fragments over 400 base pairs would likely have multiple copies of the adapters ligated to their ends.

The size selected amplicons were PCR amplified using primers to the ION torrent P1 adapter and A adapter sequences, to generate at least 1 μg of unbiased amplicons of all the barcoded-adapter-ligated DNA fragments. PCR amplifications were performed using the following parameters: (1) initial denaturing at 98°C for 30 s; (2) 8–11 cycles of 98°C for 10 s, 58°C for 30 s, and 65°C for 30 s; (3) 65°C for 5 min; and (4) a final hold at 4°C. After amplification, the amplicons in each sample were purified using Agencourt AMPure XP and the DNA concentration was determined by qPCR using the Ion Universal Library Quantitation Kit (ThermoFisher Scientific: Cat. No. A26217) according to the Ion 16S Metagenomics Kit User Guide (pgs. 18–22). Each sample was assayed in replicates of three. The calculated average concentration for each sample was used for subsequent library pooling calculations.

Each 16S barcoded library were diluted and pooled in equal molar concentrations into one of three pools: Pools A, B, and C (Table S2). Each pool was PCR amplified using the P1 and A adaptor primers from the NEBNext Fast DNA Library Prep Set for Ion Torrent with the following modified PCR parameters: (1) initial denaturing at 98°C for 30 s; (2) 30 cycles of 98°C for 10 s, 58°C for 30 s, and 65°C for 30 s; (3) 65°C for 5 min; and (4) a final hold at 4°C. After amplification, DNA in each pool was purified using the Agencourt AMPure XP. Our yield was ~1,000× higher than needed and contained a higher proportion of short fragments, which led to the need to modify the templating procedure. We created Pool D to re-sequence a subset of sample libraries which had the fewest number of reads (Table S2), described in more detail in the supplementary sequencing pool section below. For that run, we shortened the number of cycles at this amplification step to 25, significantly improving the library quality (proportion of full-length fragments). We also modified our dilution method to generate more accurate equal-molar pooling. The final concentrations of the pooled libraries were verified by qPCR using the 16S Metagenomics Kit User Guide protocol as already described.

### Template Preparation, Enrichment, and Sequencing

The first step required to sequence our DNA amplicons using the Ion Torrent platform (Thermo Fisher Scientific Inc., Waltham, MA USA) is to template each of our pooled DNA libraries. Templating involves loading appropriately diluted pooled libraries onto ion sphere particles (ISPs) using the Ion PGM Hi-Q View OT2 Kit (ThermoFisher Scientific: Cat. No. A29900) and the Ion OneTouch-2 and OneTouch-ES Instruments (Thermo Fisher Scientific: Cat. No. 4474779) according to the manufactures' protocol for 400 base pair read length templating. The manufactures' protocol for templating lists a suggested concentration for each type of library in order to acquire a target range of template positive particles. Successful templating is achieved when the sample passes a quality control test, which is explained in more detail below. The concentration of the freshly diluted pooled libraries that were successfully templated and subsequently sequenced in this study were as follows: Pools A and C at 26 pM each, Pool B at 23 pM, and Pool D at 10 pM.

To determine the percentage of ISPs with bound template DNA (pooled library), a PCR thermocycler, a Qubit 3.0 Fluorometer (Thermo Fisher Scientific: Cat. No. Q33216), and the Ion Sphere Quality Control Kit (Thermo Fisher Scientific: Cat. No.4468656) were used according to the manufactures' protocol found in the Ion PGM Hi-Q View OT2 Kit manual. The acceptable level of templated ISPs is 10–30% with an ideal range from 20 to 25%. The percent templated ISPs for each pool were determined to be as follows: 31% for Pool A; 18% for Pool B; 22% for Pool C; and 16% for Pool D. Though the percent templated ISPs for Pool A was 1% higher than the acceptable level, we have found that the percentages generated tend to have a variation of a few percentage points when performed in replicate, so we deemed Pool A to be sufficiently close to acceptable levels. Templating preparations of pooled libraries that passed the quality control assay were then enriched for template positive ISPs using the Ion OneTouch ES Instrument and Ion PGM Enrichment Beads (Dynabead MyOne Streptavidin C1 beads; Thermo Fisher Scientific: Cat. No. 4478525) which function to selectively bind to the template positive ISPs.

The Ion Torrent PGM system preparation, sequencing run, and post-run cleanup were performed using the chemistry and supplies provided in the Ion PGM Hi-Q Sequencing Kit (Thermo Fisher Scientific: Cat. No. A25592) and Ion PGM Hi-Q Wash 2 Bottle Kit (Thermo Fisher Scientific: Cat. No. A25591) according to the sequencing protocol in the Ion PGM Hi-Q Sequencing User Guide as follows. First, the sequencing run plan was created on the Ion Torrent Server with the following criteria: (1) Application—Metagenomics; (2) Target technique-−16S Targeted Sequencing; (3) Library Kit—Ion Plus Fragment Library Kit; (4) Template Kit—Ion PGM Hi-Q View OT2 Kit-−400; (5) Sequencing Kit—Ion PGM Hi-Q Sequencing Kit; (6) Flows −850; (7) Chip Type—Ion 318™ Chip v2; and (8) Barcoded Set—IonXpress. Second, the sequencing primers were annealed to the template positive ISPs using a PCR thermocycler (95°C 2 min, 37°C 2 min). Third, the sequencing polymerase was added to the primer annealed template positive ISPs and loaded onto an Ion 318™ Chip v2 (Thermo Fisher Scientific: Cat. No. 4484354). Fourth, the loaded Ion chip was placed into the cleaned and initialized Ion Torrent PGM instrument (Thermo Fisher Scientific: Cat. No. 4462921) and the sequencing run was initiated.

After sequencing Pools A, B, and C, and preliminary analyses, we found that some of the individual libraries within each pool had low numbers of mapped reads. This was not unexpected considering other studies using the equal-molar pooling method of combining multiple samples into one sequencing run have also reported widely varying numbers of read sequences generated among samples within a pool (Lemos et al., 2011). To address this potentially problematic issue, an additional sequencing run was performed. Using the following predetermined criteria, specific samples were chosen for a supplementary sequencing pool (Pool D): Samples that generated 2,500 mapped reads or less and generated a metagenomic profile with low species diversity compared to other samples of the same treatment group. The samples meeting these criteria were C31, C35, 44, 46, N43, N45, and N47 (Table S2). Though meeting the above criteria, one sample, C34, had no mapped reads despite using all of the P1/A adaptor PCR reaction when pooling (pool A), thus this sample was not reamplified. After preliminary analysis of Pool D, the new sequencing data were combined with the previous data collected for each sample.

### 16S Metagenomic and Statistical Analysis

Using the Ion Reporter Software, the sequencing data for each individual sample were organized into their appropriate test group: Baseline, PG-inoculated, sham-inoculated, or pregnant. These groups were then analyzed using a stringently designed workflow to generate the 16S Oral Microbiome Profiles. The workflow included the following criteria: (1) Application—Metagenomics; (2) Sample Groups—Single/Multi; (3) References—Curate MicroSEQ® 16S Reference Library v2013.1 and Curate Greengenes v13.5; (4) Primers—Default; (5) Primer(s) Detected—Both ends; (6) Minimum Alignment Coverage −90.0%; (7) Read Abundance Filter −10 copy minimum; (8) Genus Cutoff −97.0%; (9) Species Cutoff −99.0%; and (10) Slash ID Reporting Percentage −0.2%. This workflow was used to map the sequencing data (i.e., assign reads to Operational Taxonomic Units) for each individual sample, as well as the collective (consensus) data for each group, against multiple curated reference microbial genome databases to generate individual microbiome population profiles (OTU data sets) for each sample as well as consensus profiles for each treatment group. The similarity BP (base-pair) cutoff was set to 150 for all data reported here. Each individual sample was also analyzed to determine if there were any microbiome profiles that appeared unusual or as outliers when compared to the rest of the profiles within their experimental group. The consensus microbiome profiles were then compared between treatment groups and/or time points to identify shifts in the microbial population in response to *P. gingivalis* inoculation or pregnancy and for analyses of other descriptors associated with the identified community members at the family, genus, or species level (i.e., Gram stain characteristic, oxygen requirements, predictive metabolic profiles). R software (R Core Team, 2013) was used to graphically display the population percentage of identified community members within in each microbiome profile in context of the selected group descriptor.

The mapped output data (microbiome population profiles) for each treatment group were assessed and graphically displayed by performing alpha and beta diversity analysis using the QIIME bioinformatics pipeline (Caporaso et al., 2010) on the Ion Reporter Software Suite (Thermo Fisher Cloud). These analyses produce descriptive community statistics. We report alpha diversity intra-group analysis of the consensus microbiomes, comprising of all samples belonging to a group, for each of our four groups. The alpha diversity pipeline generates rarefaction curves which reflect the species (community) abundance (number of OTUs) and evenness (population proportion) within the group, and thus if the sequencing data output was deep enough to accurately characterize the microbiota present at the time of sampling they will generate rarefaction curves (phylogenetic diversity plots) that plateau when only the rarest species remain to be sampled. Four different nonparametric statistical analyses were completed to create rarefaction curves: (1) Observed Species, (2) Simpson index, (3) Shannon index, and (4) Chao1 index. The Observed Species and Choa1 analyses measure sample/community richness. Simplistically, the output based on these measures depends on the presence or absence of species observed in samples within a group. Chao1 index also reflects population proportion (evenness) to a degree by taking in account the number of times a species occurs. However, these methods are strongly affected by sample size, which can lead to an underestimation of sample richness (Colwell and Coddington, 1994), and thus are not as popular a measure when performing analyses on 16S metagenomic data generated using short read platforms like Ion Torrent PGM or MiSeq when the number of OTUs identified are low in a study. That said, after reaching plateau, the estimation of diversity becomes relatively independent of sample size (Hughes et al., 2001). Moreover, using six 16S regional targets rather than one or two would compensate for small sample size leading to poor coverage common to 16S metagenomic analysis performed on these small read platforms that commonly use only one or two regional targets. Using six regional targets would also diminish the occurrence of false positive that reportedly (Edgar, 2017) plagues QIIME analysis of 16S metagenomic data generated from short read platforms when using a single variable region target. The Simpson and Shannon analyses reflect population diversity (abundance and evenness). More precisely, the Simpson index reflects species dominance and the probability of two individual organisms that belong to the same species being randomly chosen. Similar to the Chao1 index, it is weighted toward the abundance of the most common species. For the Simpson Index, a measure of 1 indicates complete evenness in species proportions within that sample or group. In contrast, the Shannon Index measures the average degree of uncertainty in predicting as to what species an individual organism will belong when chosen at random, thus this measure is affected by both the number of species identified and their population proportion (evenness).

Bray-Curtis beta diversity analyses was used to compare individual sample or group microbiome profiles to each other to identify community dissimilarities. This is a distance based statistical analysis used to identify sources of variance within individual sample communities or among groups. This method uses abundance of an OTU but not the phylogeny it belongs to, unlike the UniFrac method to estimate beta diversity. To visualize beta diversity, Principal Coordinate Analysis (PCoA) plots were used. PCoA is a multidimensional scaling computation that converts the microbiome population profile data into correlated similarities variables, then transforms them into a dissimilarity or distance matrix that can then be spatially plotted on a set of three orthogonal axes as a data point such that a maximum amount of variation for that data point is explained by the first principal coordinate axes, the second largest amount of variation is explained by the second principal coordinate axes, etc. (Caporaso et al., 2010).

### Predicted Microbial Metabolome Profiles

A predictive analysis of morphological classification, oxygen requirement, and major metabolite synthesis profiles was performed. To identify the capacity to produce major metabolites in the oral cavity in response to *P. gingivalis* inoculation and then pregnancy, the consensus microbiome profiles were analyzed using KEGG mapper (www.genome.jp/kegg/) and further reviewed using the Substrata database (www.datapunk.net/substrata) and STRING (string-db.org). In order to be classified as belonging to a select metabolite group, the metabolite must be identified as a major end product rather than just having the genetic capacity to generate the metabolite in question. Bergey's manual of systematics of archaea and bacteria was also used to support appropriate metabolite classification of each genus (Whitman, 2015). When classification was unclear due to lack of strong evidence to support inclusion, or if strong evidence supported being excluded as a major producer of any one of these select metabolites, these identified members were classified as Other. All predictive qualitative metabolic outcome designations were plotted as percent OTU at the genus level.

## Results

### Impact of *P. gingivalis* Infection on Alveolar Bone Loss and Humeral Immunity

**Figure 1:**
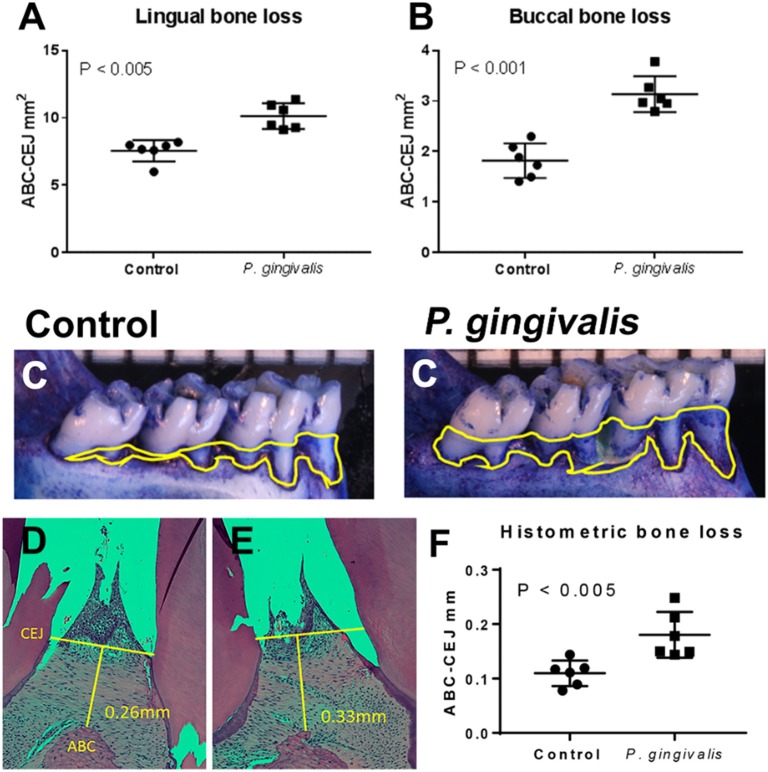
All *P. gingivalis* inoculated animals had significantly greater alveolar bone loss than aged-matched sham-inoculated controls (baseline) confirming that these animals had periodontal disease. Morphometric assessment of mandibular and maxillary bone loss in pregnant control and PG-inoculated rats. Values in each graph represent the extent of horizontal bone loss, labeled as CEJ-ABC area measurements (mm^2^), on the lingual **(A)** and buccal **(B)** sides from each mandible. **(C)** Representative images of the lingual aspect of the rat mandible illustrating the extent of horizontal bone loss. Yellow lines demarcate the CEJ-ABC junction that was used to determine extent of bone loss. Representative images of maxillary interdental papilla from control **(D)** and PG-inoculated rats **(E)** demonstrating how CEJ and ABC measurements were taken for histomorphometry **(F)**. Bars in all graphs show the mean ± SD. Data were analyzed by unpaired student's t test.

All baseline and sham-inoculated samples were negative for *P. gingivalis* by PCR. All specimens from the PG and pregnant groups were positive for *P. gingivalis* by PCR. All *P. gingivalis* inoculated animals had significantly greater alveolar bone loss than aged-matched sham-inoculated controls confirming that these animals had periodontal disease (Figure 1).

**Figure 2:**
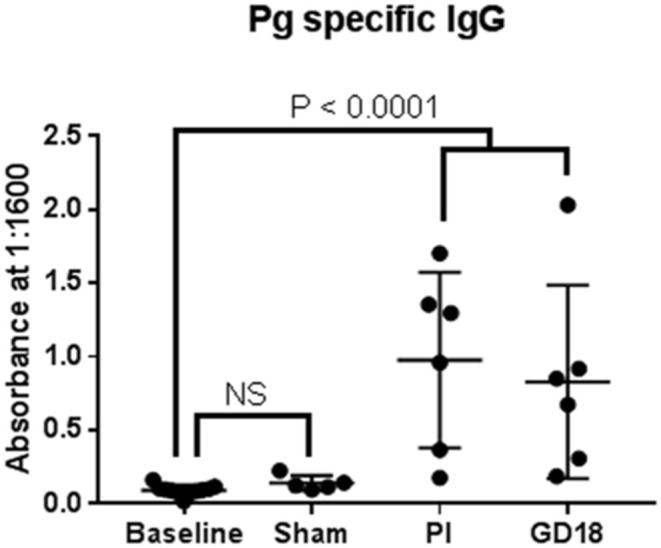
We also measured *P. gingivalis* specific IgM and IgG before (baseline), after inoculation, and during pregnancy to assess microbial exposure.

We also measured *P. gingivalis* specific IgM and IgG before (baseline), after inoculation, and during pregnancy to assess microbial exposure (Figure 2). None of the animals had detectable *P. gingivalis* specific IgM at any time point in the study (data not shown). There was no difference in the level of *P. gingivalis* specific IgG between baseline and sham control groups. Both PG inoculated and pregnant groups had greater amounts of *P. gingivalis* specific IgG than the baseline group (*P* < 0.0001).

### The Effect of *P. gingivalis* Infection and Pregnancy on the Oral Microbiome

We first looked for shifts in the native oral ecology by evaluating the intra-group richness, diversity, and relative depth of coverage at each collection time point and condition. Sequences (reads) with more than 10 copies (abundance) and a sequence match that mapped at 90% or greater accuracy to the reference genomes are assigned a specific OTU. These are standard criteria to maximize the confidence that the identified bacteria are all significant contributors to microbiome community. For our data, the specific mapping cutoff was 97% sequence match at the genus level and 99% sequence match at the species level for all qualifying reads. All specimens, with the exception of one sham-inoculated control sample (ID# 34), generated OTUs that met these criteria. To visualize our data, consensus microbiome profiles, graphed as OTUs at the family level are shown in the Figure S1. Consensus microbiome profiles at the family, genus, and species levels, sorted by 16S variable region, were also visualized using Krona charts generated using the Ion Torrent software suite and are shown in the Figures S2, S3.

**Figure 3:**
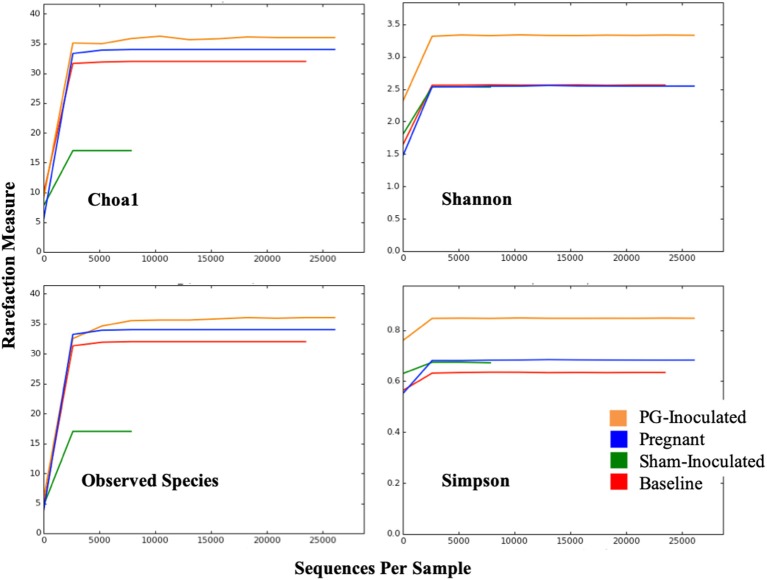
Alpha diversity was evaluated by four different statistical analyses including: Observed Species, Simpson Index, Shannon Index, Chao1 index.

Alpha diversity was evaluated by four different statistical analyses including: Observed Species, Simpson Index, Shannon Index, Chao1 index (Figure 3). The rarefaction curves plateaued for all groups, indicating that our data sets had adequate coverage and only the rarest species may not have been identified. Both Observed Species and Choa1 rarefaction curves showed that abundance was similar among PG-inoculated, pregnant, and baseline groups, while the sham-inoculated group, which had the lowest number of OTUs, had the least abundance. In contrast, the Shannon and Simpson rarefaction curves, which also reflect population evenness, showed that PG-inoculated group had the greatest diversity. Specifically, the average Shannon and Simpson diversity indices at the species level were, respectively, 3.24 and 0.84 (PG-inoculated), 2.45 and 0.67 (pregnant), 2.47 and 0.63 (baseline), and 2.35 and 0.66 (sham-inoculated), where a higher value indicates greater diversity in that group.

**Figure 4:**
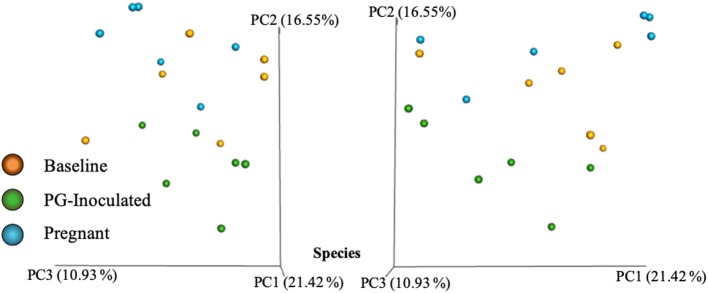
Bray Curtis PCoA plot of beta diversity measures of dissimilarity shows at the species level, baseline, PG-inoculated, and pregnant groups clearly clustered within their group along PC2 not PC1.

**Figure 5:**
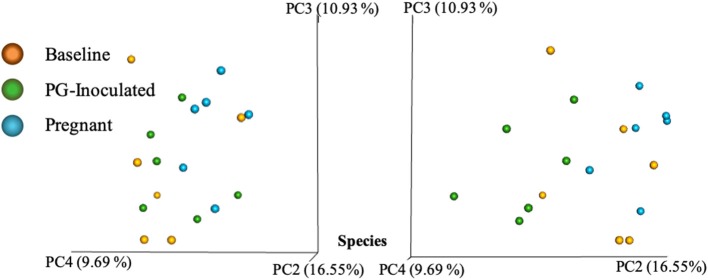
Bray Curtis PCoA plot of beta diversity measures of dissimilarity shows at the species level, baseline, PG-inoculated, and pregnant groups clearly clustered within their group along PC2 and PC4 but not PC3.

**Figure 6:**
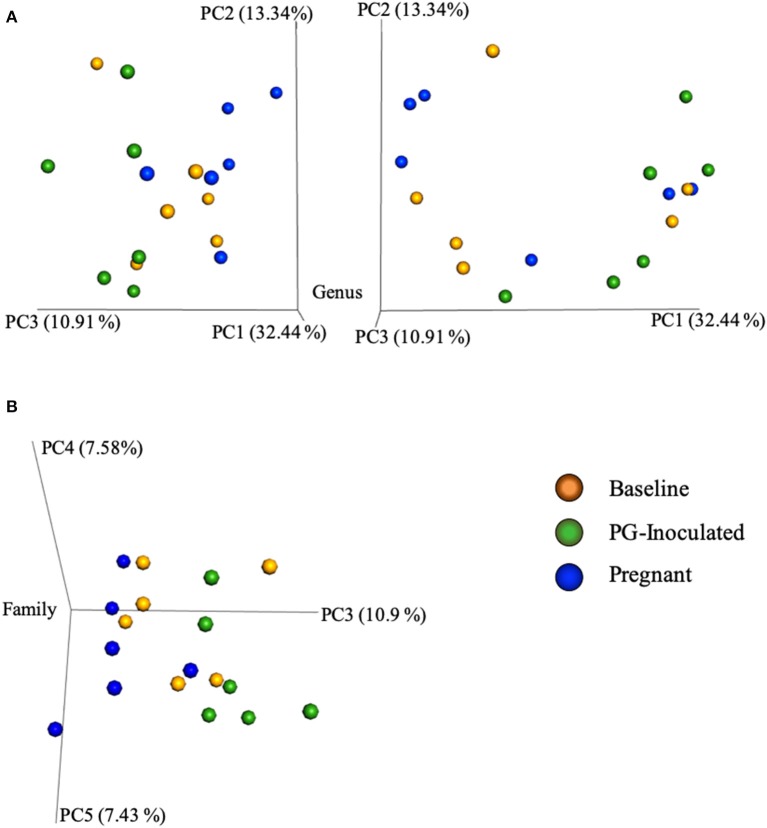
Bray Curtis PCoA plot of beta diversity measures of dissimilarity shows that at the genus level, the baseline, PG-inoculated, and pregnant groups clustered within their groups along PC3 at the **(A)** genus level and **(B)** family level.

**Figure 7:**
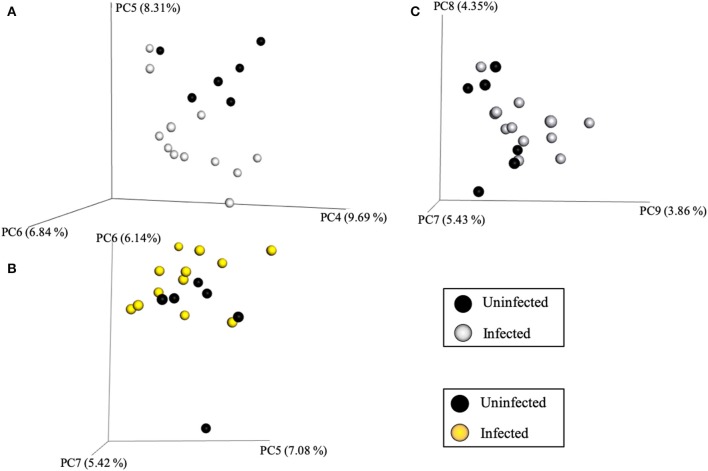
Bray Curtis PCoA plot of beta diversity measures of dissimilarity shows, when the data sets were sorted by infection status, they clustered within their groups along PC5 at the species level **(A)**, PC6 at the genus level **(B)**, and PC9 at the family level **(C)**.

Community variance was visualized though 3-dimensional Bray Curtis PCoA plots. Each data point was sorted and color coded by experimental group, oral infection status, or pregnancy status, and plotted across three primary variation distance measures. The lack of clustering along PC1 indicated that experimental treatment was not the primary source of variation (Figure 4). However, experimental groups did contribute to community variance at the family, genus, and species level. For example, at the species level, baseline, PG-inoculated, and pregnant groups clearly clustered within their group along PC2, which accounted for 16.55% of community variance (Figure 5). These distance measures data also showed that the pregnancy group was positioned most distant from zero along PC2, indicating that pregnancy in each infected animal exerted a greater degree of oral microbial community variance than *P. gingivalis* infection alone at the species level. At the genus and family level, the baseline, PG-inoculated, and pregnant groups clustered within their groups along PC3: 10.91% at the genus level and 10.90% at the family level (Figure 6). When sorted by infection status (Figure 7), the data sets clustered within their groups along PC5 at the species level (8.31%), PC6 at the genus level (6.14%), and PC9 at the family level (3.86%).

**Figure 8:**
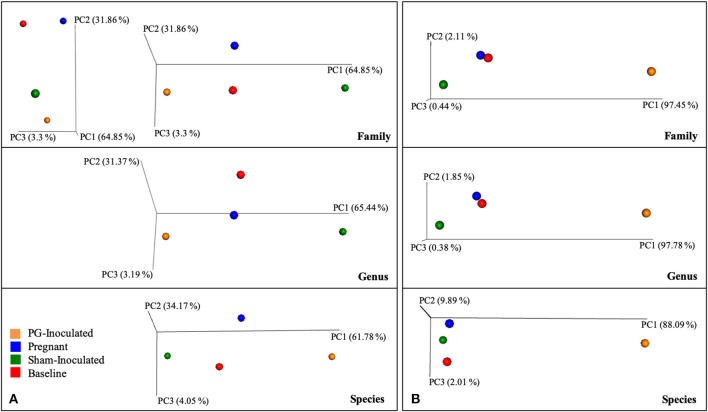
Collective consensus microbiome at the family, genus, or species level and plotted as four data points in PCoA plots using **(A)** Bray Curtis or **(B)** Eucledian of beta diversity measures of dissimilarity.

Beta diversity analysis was also performed to determine group dissimilarity between each collective consensus microbiome at the family, genus, or species level and plotted as four data points in PCoA plots. By merging data sets within each group, more rare members identified in each sample have less impact on the distance measures, thus generating plots in an alternative context, similar to alpha diversity measures. Example graphs are shown in Figure 8. When Bray Curtis metrics were used and the data points were visualized along the first three axes of variation, both baseline and pregnant group plotted positions were found close together while PG-inoculated and sham-inoculated were both positioned away. Though PG-inoculated and sham-inoculated clustered together along PC2, they were the most distantly plotted points from each other when viewed with respect to PC1. In contrast, when Euclidian metrics of community dissimilarity were used, which only take in account presence-absence and not abundance, sham-inoculated, baseline, and pregnant microbiomes clustered closely together while PG-inoculated microbiome plotted distantly.

### Similarities and Differences in the Oral Microbial Composition in Healthy Non-pregnant Uninfected Rats

Specimens collected prior to inoculation (baseline) as well as specimens collected from sham-inoculated animals were used as uninfected controls for this study. Baseline profiles represent the normal oral flora in 2-month old female rats prior to antibiotic treatment, whereas the sham-inoculated group represents age-related 5-month old socially mature rats previously treated with antibiotics and sterile vehicle. When comparing the microbiome profiles between baseline and sham-inoculated groups, the two dominant families identified were *Pasteurellaceae* and *Streptococcaceae* (Figure S1). *Pasteurellaceae* made up 39.55% of the population observed for the baseline group, and 44.32% of the population in the sham-inoculated group. The dominant species identified within the *Pasteurellaceae* (75–85%) was *Haemophilus parainfluenzae* at 75–85%.

Notable differences at the family level between the sham and baseline groups were a relatively larger population of *Lactobacillaceae* in the sham-inoculated group (15.81%) compared to the baseline group (1.13%) and the relatively larger population of *Staphylococcaceae* in the baseline group (43.56%) compared to the sham group (17.83%). A lesser but notable difference was also observed for *Veillonellaceae* (8.14% sham-inoculated; 2.95% baseline) and *Micrococcaceae* (0.37% sham-inoculated; 6.20% baseline).

Roughly half of all mapped reads (OTUs) in each test group derived from the same animals could be identified down to the species level (i.e., 50.0% of baseline; 54.6% of PG-inoculated; 58.0% of pregnant) while a larger percentage of Sham-inoculated OTUs could be mapped to the species level (68.8%).

It was difficult to evaluate changes in the *Streptococcus* at the species level among all groups due to the high level of species level slash calls for this genus (Figures S2, S3). Slash calls are sequences (OTUs) that aligned with more than one possible reference sequence, and where the percentage difference between these top hits is ≤0.2% of the sequence. For example, only 11.04% of the *Streptococcus* population could be further identified to the species level in the baseline group while 53.73% of the *Streptococcus* population in the sham-inoculated group could be identified to the species level. However, we could specifically review select species, for example: 3.52% of the microbial population in the baseline group and 1.67% in the sham-inoculated group were identified as *Streptococcus sanguinis*. Overall, our data illustrates that *Streptococcus* species are typically diverse in the rat oral cavity and that the majority of the OTUs that mapped to *Streptococcaceae* family are currently unmappable to the species level.

### Similarities and Differences in the Oral Microbial Composition in Healthy Non-pregnant Rats in Response to *P. gingivalis* Inoculation

To determine *P. gingivalis*-induced changes in the normal oral microbiome, we compared their consensus microbiome profiles collected post-inoculation (PG-inoculated) to samples collected prior to infection (baseline). We found that *Porphyromonadaceae* was present in the PG-inoculated group, albeit a at 0.02% of the total mapped microbiome population, confirming animals were indeed colonized.

At the family level (Figure S1), *Streptococcaceae* and *Pasteurellaceae* were the most dominant; however, the proportion of *Pasteurellaceae* decreased after *P. gingivalis* inoculation from 39.5% at baseline to 14.4% in the infected non-pregnant animals (PG-inoculated). In contrast, the *Streptococcaceae* population slightly increased from 43.6% at baseline to 55.4% in the PG-inoculated group.

Again, comparative analyses of *Streptococcus* species were incomplete due to the high level of slash calls that mapped to this genus (Figures S2, S3). Nevertheless, our data indicate that the population proportion distribution of *Streptococcus* species altered in response to *P. gingivalis* colonization. Specifically, we identified the following species in the baseline group, in order of decreasing population proportion: *S. sanguinis, S. mutans, S. hyointestinalis, S. infantis, S. oralis, S. australis, S. danieliae, S. suis, S. lactarius*, and *S. pneumoniae*. Whereas, the species that could be specifically identified in the PG-inoculated group, in order of decreasing population proportion, were *S. mutans, S. sanguinis, S. infantis, S. oralis, S. lactarius, S. australis, S. sinensis, S. pneumoniae, S. merionis, S. parasanguinis, S. anginosus*, and *S. pseudopneumoniae*.

Other changes noted in the PG-inoculated microbiome profile were an increase in proportion of *Proteus mirabilis* and *Enterococcus faecalis* species as compared to controls. Overall, these data suggest that *P. gingivalis* inoculation perturbed the oral microbiome, leading to a shift in the microbiome profile with increased microbial diversity, which corroborates with our alpha and beta diversity statistical analyses.

### Shifts in the Oral Microbial Composition of *P. gingivalis* Infected Rats in Response to Pregnancy

In order to evaluate the interaction of pregnancy and periodontal disease, we compared the consensus oral microbiome profiles prior to inoculation (baseline), at the end of the inoculation phase (PG-inoculated), and at GD 18 (pregnant). As in the baseline and PG-inoculated groups, *Streptococcaceae* and *Pasteurellaceae* remained the two most dominant families in the pregnant group microbiome (Figure S1). There were however, some distinctive differences and notable shifts in the pregnant group. For example, *Streptococcaceae* and *Pasteurellaceae*, appear to be reverting toward baseline levels. A similar trend was observed among the other less common families with the following notable exceptions. First, *Porphyromonadaceae*, which was present only in PG-inoculated and pregnant groups, increased from 0.02% in the PG-inoculated group to 0.74% in the pregnant group. Second, *Corynebacteriaceae* population notably increased to 13.49% in the pregnant group microbiome from 1.93% in the baseline group and 3.13% in the PG-inoculated group.

At the species level (Figures S2, S3), *Haemophilus parainfluenzae* rebounded and became the dominant single species identified in the pregnant group. Similarly, many of the minor species that appeared in the PG-inoculated time point reverted to near baseline levels during pregnancy. Notable exceptions included *Corynebacterium mastitidis* which made up 98.96% of identified *Corynebacterium* species. Again, changes in *Streptococcus* at the species level could not be fully assessed due to the high level of slash calls. The fraction of the population within the *Streptococcus* genus that could be positively identified down to the species level in the pregnant group was 10.78%, which was similar to the 11.04% at baseline, but less than the 47.49% of the PG-inoculated group. Though a large proportion of *Streptococcus* OTUs could not be mapped down to single species, the standardized methodology used prohibits group specific bias so species comparisons among groups are valid. To illustrate how consistent the data was, allowing group profile comparisons, we assessed relative population proportions of *S. mutans* relative to all OTU categories. For example, *S. mutans* made up 2.18% at baseline, 28.61% at PG-inoculated, and 2.75% at pregnancy of all *Streptococcaceae* family OTUs. These numbers correlate with the population proportion of *S. mutans* relative to all identified species level OTUs (1.83% baseline; 29.05% PG-inoculated; 1.63% pregnant), as well as the percent proportion of all OTUs (0.91% baseline; 15.85% PG-inoculated; 0.95% pregnant) that mapped to any microbial family. Overall, this genus appears to be consistently diverse with *S. sanguinis, S. mutans*, and *S. infantis* being the most dominant members identified.

### Metagenomic Shifts Relative to Morphological Classification, Oxygen Requirement, and Major Metabolite Profiles

**Figure 9:**
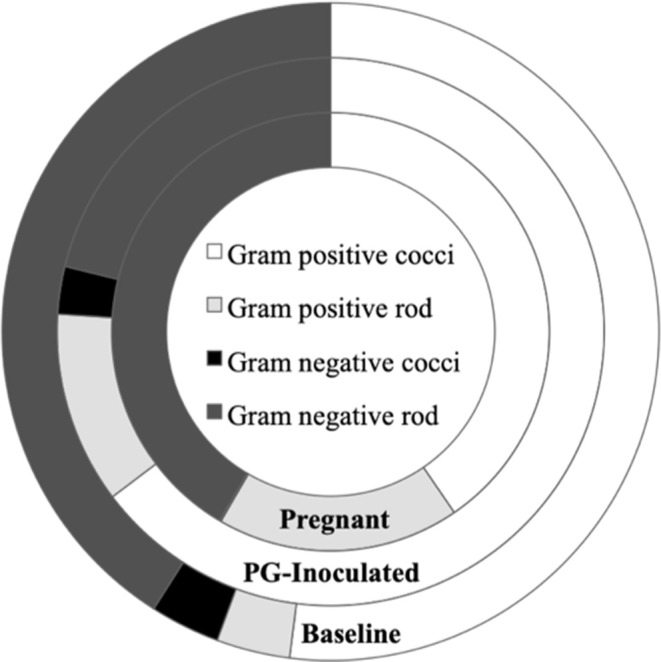
Population proportion of identified community members in terms of predicted Gram stain classification and cell morphology for baseline, PG-inoculated, and pregnant groups.

In addition to assessing the changes in microbiome profiles in terms of microbial identity, we also performed predictive analyses of select microbial cellular functions. The oral microbiome has classically been sorted by morphological characteristics and Gram's classification so the first step in our functional analysis was to sort our consensus data in these terms. Though no spirochetes were detected, shifts were observed in the consensus profiles of Gram-positive and Gram-negative bacteria (Figure 9). For example, when the conditions in the host shifted from baseline to PG-inoculated, there was an increase in Gram-positive bacteria, most notably, there was over a 3-fold increase in Gram-positive rods and a 2-fold decrease in Gram-negative rods. The pregnant group showed an even larger increase in Gram-positive rods, reflecting an almost 5-fold increase relative to baseline. In contrast, the population proportion of Gram-negative rods shifted back toward baseline levels during pregnancy. The population proportion of Gram-negative cocci was similar between the baseline and PG-inoculated groups, but decreased from ~3% down to 0.1% in the pregnant group. The observed changes become more interesting when the oxygen requirements of the detected members of the consensus population were considered in our following analysis.

**Figure 10:**
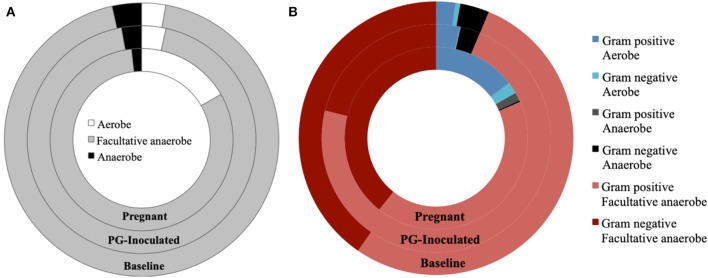
Population proportion of identified community members in terms of predicted **(A)** oxygen requirements alone, or **(B)** oxygen requirements in context of Gram stain classification, for baseline, PG-inoculated, and pregnant groups.

We then profiled microbial populations based on their oxygen requirement (Figure 10A). While the proportion of facultative anaerobic bacteria remained relatively constant between baseline and PG-inoculated, the proportion of strictly anaerobic bacteria shifted down and the proportion of aerobes shifted up by 0.6% of the total population (Figure 10B). In contrast, the population proportion of facultative anaerobes shifted down from 93.7 to 81.5% in response to pregnancy. This change was accompanied by a decrease in strict anaerobes (2.8–1.7%) and an increase in strict aerobes (3.4–16.8%), reflecting an overall increase in oxygen requirement of the oral microbiota.

**Figure 11:**
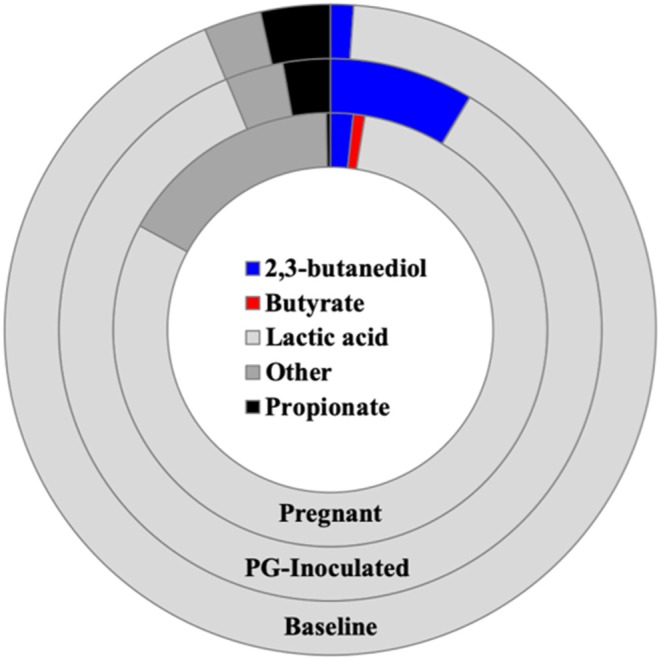
Population proportion of identified community members in terms of predicted metabolic characteristics as a major generator of target microbial metabolites for baseline, PG-inoculated, and pregnant groups.

**Figure 12:**
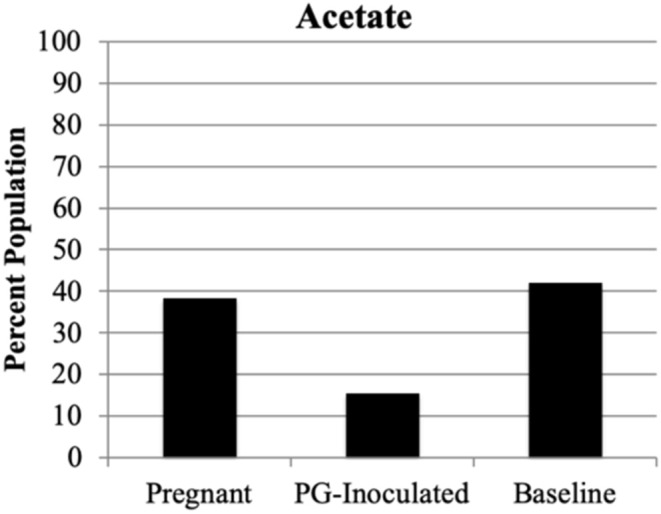
Population proportion of identified community members in terms of predicted metabolic characteristic as a major generator of acetate for baseline, PG-inoculated, and pregnant groups.

We next profiled the predicted microbial metabolomes of all groups. Among the wide variety of metabolites that could be identified, 2, 3-butanediol, butyrate, lactic acid, and propionate were found to be the most population defining products by which most members of each microbiome could be differentially sorted. *Anaerostripes* is the only genus that we determine should be categorized as belonging to both the butyrate and lactic acid major metabolite groups. All others could be sorted into one of these four metabolite groups or placed into the “Other” group as not a major producer of these metabolites (Figure 11). The predicted population proportion of lactic acid producers decreased from 92.57% (baseline) to 85.15% (PG-inoculated) to 80.50% (pregnant). The proportion of propionate producers decreased from 3.44%, (baseline) to 2.81% (PG-inoculated) to 0.30% (pregnant). In contrast, the proportion of butyrate producers progressively increased from 0%, (baseline) to 0.02% (PG-inoculated) to 0.86% (pregnant). The proportion of 2, 3-butanediol producers increased from 1.15% (baseline) to 8.59% (PG-inoculated) in response to PG-infection but then decreased to 1.70% during pregnancy. The remaining population portion of bacteria designated as “Other” increased over 5-fold during pregnancy, reflecting the increase in obligate aerobes in the oral cavity of pregnant rats at GD 18. The ability to produce acetate as a major metabolite was also assessed but plotted independently because it was typically co-produced with one of the select four metabolites (Figure 12). The proportion of acetate producers decreased from 42.03% (baseline) to 15.34% (PG-inoculated) but increased in response to pregnancy to 38.38%.

**Figure 13:**
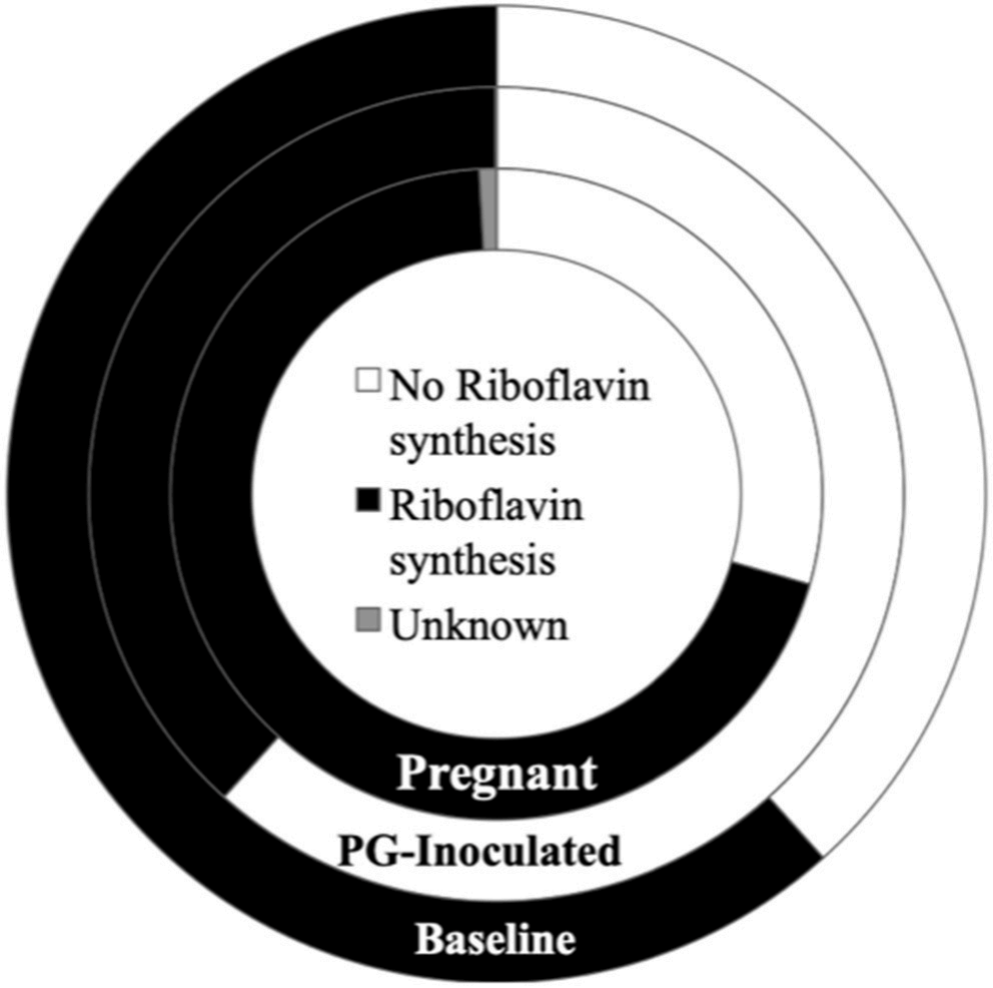
Population proportion of identified community members in terms of predicted ability to synthesize riboflavin for baseline, PG-inoculated, and pregnant groups.

Because the ability to synthesize riboflavin (Vitamin B2) is associated with gastrointestinal (GI) health (Yoshii et al., 2019) and many of the species identified in the oral cavity also colonize the GI, we performed an independent assessment of riboflavin producers within the consensus microbiome profile of each group (Figure 13). Riboflavin biosynthesis capability was determined by the genomic presence of the complete pathway, using the KEGG pathways database. The oral microbial population's predicted collective capability to synthesize riboflavin dramatically decreased from 85.4% (baseline) to 43.1% (PG-inoculated) in response to infection, but this ability recovered during pregnancy by GD 18, increasing to 95.8% (pregnant). Notably, a small proportion of the population (~1% of the pregnant group and 0.1% of the PG-inoculated group) could not be positively or negatively categorized due to limitations in the reference KEGG database at the time of analysis. A cursory analysis of all groups showed that ≤0.2% of each consensus microbiome profile had the predicted ability to feed into the porphyrin metabolism pathway, using riboflavin as an early precursor metabolite, to ultimately synthesize vitamin B12 (data not shown).

## Discussion

### The Oral Microbiome of Healthy Rats Compared to Previously Published Human and Rat Profiles

The Forsyth Institute created a publicly available human oral microbiome database (www.homd.org). Using whole 16S rRNA gene sequencing, they reported that 96% of the human oral microbiome consists of six phyla (*Firmicutes* 36.7%, *Bacteroidetes* 17.3%, *Proteobacteria* 17.1%, *Actinobacteria* 11.6%, *Spirochaetes* 7.9%, *Fusobacteria* 5.2%) (Dewhirst et al., 2010). In contrast, we found that the bacteria we identified at baseline in the oral cavity of rats belong to only three phyla (*Firmicutes* 48.10%, *Proteobacteria* 43.64%, *Actinobacteria* 8.26%). The undetected phyla of *Bacteroidetes, Spirochaetes*, and *Fusobacteria* include key periodontopathogenic bacteria associated with human periodontal disease and implicated in adverse pregnancy outcomes (e.g., *P. gingivalis, Treponema denticola, Tannerella forsythia, Fusobacterium nucleatum*) (Aagaard et al., 2014; Prince et al., 2016; Lin et al., 2018). After *P. gingivalis* inoculation, the consensus oral microbiome included four phyla (*Firmicutes* 72.22%, *Proteobacteria* 24.61%, *Actinobacteria* 3.15%, *Bacteroidetes* 0.02%) with the family *Porphyromonadaceae* making up 100% of the identified *Bacteroidetes* phylum population, consistent with experimentally induced oral infection. By GD 18, the orally infected pregnant rat oral microbiome consisted of the same four phyla, but with notable population shifts (*Firmicutes* 41.69%, *Proteobacteria* 40.11%, *Actinobacteria* 15.54%, *Bacteroidetes* 2.66%). The *Bacteroidetes* phylum in the pregnant group now consisted of four families, including *Porphyromonadaceae, Bacteroidaceae, Cytophagaceae*, and *Flavobacteriaceae*.

We found that the most abundant genus in the oral cavity of our rat model at baseline was *Streptococcus*, similar to the reported findings of the human oral microbiota (Dewhirst et al., 2010). With regards to the two most abundant genera at baseline, *Streptococcus* and *Haemophilus* are known to specifically adhere to each other (co-aggregate) during biofilm development due to outer membrane adhesions found on some *Streptococcus* species that recognize specific polysaccharide receptors on *Haemophilus parainfluenzae* (Lai et al., 1990; Heller et al., 2016). Interestingly, the large percentage of highly diverse *Streptococcus* species and the high percentage of *Haemophilus parainfluenzae* observed in these rat oral microbiomes was also observed in the development of early human dental biofilm (Heller et al., 2016), suggesting that the periodontitis and pregnancy rat model used in this study could be considered an appropriate translational model for oral pathology as it represents a relatively similar oral flora to that of a human, but with the notable absence of periodontopathogenic bacteria. Rather than a shortcoming, we view this absence as a critical advantage of this model in that it allows controlled colonization of the oral cavity with periodontopathogenic bacteria to investigate their role in oral and extra-oral disease development.

Due to few previously completed studies in the area of oral microbiomes of rats, it was difficult to compare our results to other published data. The only study we believe to be somewhat equivalent to ours for comparison was the Manrique et al. study that characterized the normal oral flora of healthy Sprague-Dawley rats by collecting the supragingival plaque of the upper molars (Manrique et al., 2013). However, the Manrique study found that *Rothia* dominated the oral cavity at 74.43% and the next most abundant bacterial genus were *Streptococcus* (4.67%). Though OTUs that mapped to the *Micrococcaceae* family was identified in all of our experimental groups (6.2–0.02%), *Rothia* was not universally found in our study. *Rothia* OTUs were found in the baseline consensus microbiome of our study at a very low proportion (1.4% total oral microbial population). The proportion found in the other groups were even lower, with 0.5% in the pregnant group and 0% (undetected) in both the PG-inoculated and sham-inoculated groups. Instead, our study found that the two most dominant genus at baseline, representing normal oral floral of healthy rats, were *Streptococcus* (41.77%) and *Haemophilus* (32.69%).

Though microbiome profiles reflect population variation among different animal groups, the observed differences between studies are also impacted by differences in animal colonies, sampling technique and differences in microbiome profile generation analysis parameters, such as stringency criteria and the reference databases used. We believe a major source of variation between our outcomes relative to recent publications are the differences in how the 16S libraries were generated. Most published studies that have reported loci specific 16S microbiomes of rats typically use sequencing libraries targeting only one or two variable regions of the 16S rRNA gene. The Manrique et al. study, for example specifically targeted the 5-6 variable region of the 16S rRNA gene, while we had more complete coverage of the target gene by sequencing seven of the nine variable region amplicons in each library (Manrique et al., 2013). Although they had a larger depth of read by using the 454 pyrosequencing platform, and thus were likely able to identify very rare species (Manrique et al., 2013), we had much better coverage of the target gene. Targeting seven sites of the 16S gene generates microbiome profiles that are reportedly comparable to full length 16S gene sequencing microbiomes, while targeting a single or only a few variable regions results in incomplete microbiome profiles (Bolchakova et al., 2015). Our data supports this observation when we viewed primer specific microbiome profiles generated from our data sets. For example, when viewing the consensus baseline microbiome profile with respect to each primer set, OTUs that could positively map to *Rothia* species only occurred for sequences derived from the V4, V6-7, and V8 primers among the seven sets used. Similarly, we found that *Streptococcaceae* dominated the oral microbiome profile (66.74–85.29%) when using the same technique in Sprague Dawley rats (Reyes et al., 2018). This suggests that our consensus profiles more accurately reflect the population proportions of the identified family, genus, and species than studies using only one or two variable regions for analysis.

### Oral Community Diversity in Pregnant Rats With Periodontal Disease Was Relatively Similar to Uninfected by GD 18 in Terms of Abundance and Evenness

While the relative diversity of an individual's microbiome does not necessarily indicate disease (He et al., 2015), there is reported evidence that the oral microbiome from patients with periodontal disease typically display greater diversity (Griffen et al., 2012; He et al., 2015), while data reported for other diseases such as data reported for dental caries typically display a reduction in diversity as a consequence of an increase in abundance of certain species in the flora isolated from the affected sites that are contributing to pathology (He et al., 2015). Among methods used to determine diversity, alpha diversity measures that take into account both abundance and evenness are reported to be better predictors of biologically relevant diversity (McCoy and Matsen, 2013). In our study, the Shannon and Simpson indices seem to indicate that the alpha diversity between the two control groups (baseline and sham-inoculated) were very similar. However, the Shannon and Simpson diversity indices estimated for the pregnant group was likewise similar to the controls. In other words, collectively, the population proportion of positively identified species were similar between pregnant, baseline, and sham-inoculated groups, despite sham-inoculated group having the fewest number of positively identified species, while PG-inoculated group had the greatest diversity. Considering the pregnant animals had periodontal disease, as indicated by their level of alveolar bone loss, our data illustrates the limitations of using alpha diversity analysis as a strict independent indicator of disease. A possible explanation for our observations that would be consistent with previous reports is that by GD 18, periodontal disease progression may have arrested and was on a trajectory of healing and perhaps the diversity indices reflect a state in which the oral microbiome is shifting back toward health.

While alpha diversity rarefaction curves reaching plateau reportedly indicate that that all but the rarest species were identified, this interpretation is based on some assumptions with respect to sample collection and processing. Due to some uncertainty that there was enough template sequenced for some of our libraries to comfortably claim that the data collected accurately represent the oral community found since there were differences between the number of reads each sample generated, we chose to create Pool D for a subset of samples identified as having the fewest number of reads from Pools A, B, and C. When comparing the data sets generated from each repeated sample, we found that their individual microbiome profiles, as analyzed by Bray Curtis beta diversity and visualized in PCoA plots, remained tightly clustered to each other and within their experimental group, thus all consensus data reported here included all the collected sequence data from all four pools A–D. This outcome also supports our belief that our samples, including those samples that had the fewest OTUs after their first sequence run in pools A–C, were of adequate sample size to create representative profiles of the microbiome found in the oral cavity of each animal, within the limits of the reference databases used.

### Oral Community Diversity in Terms of Species Composition Dissimilarities Were Conditionally Dependent, Shifting in Response to Both PG-Inoculated and Pregnancy

The advantage of using beta diversity analysis, as our approach to community analysis, is that PCoA allows discovery of the most important axes along which samples vary (dissimilarity). When using an appropriate beta diversity metric, PCoA plots also identifies similar samples as clustered points along an ax(s) of variance through visual examination of the plots. Bray-Curtis dissimilarity was selected because it is one of the most well-known approaches to quantify the difference (distance) between samples. This metric is also based on OTU abundance rather than just presence-absence data; thus the “size” and “shape” of the count vectors are taken into account in this statistical measure, which ranges from zero to one with zero defined as identical. Because the potential sources of variance (e.g., variables of interest) are not pre-defined before performing this type of statistical analysis, identifying the source of the data variance (why they cluster) may not be obvious. It can be particularly challenging for complex, multifactorial, non-categorical, or longitudinal data.

Intra-animal Bray-Curtis beta diversity analysis between baseline, PG-inoculated, and pregnancy appear widely scattered along PC1 when each animal's microbiome was plotted individually (Figure 4), but when the consensus data of each group was plotted as single points, this metric suggests that *P. gingivalis* infection was the greatest source of population variation in this study (Figure 8). Each individual sample point from baseline, PG-inoculated, and pregnant groups did clearly cluster within their groups along the PC2 axis at the species level, indicating that collectively, the experimental group conditions were the second greatest source of species variation (Figure 4). At both the genus and family level, however, the ability to distinctly discriminate between experimental groups shifted from PC2 to PC3, indicating that the core population proportions of member family and genus were more resistant to perturbations at each collection time-point over the course of the study than were specific species identity. Together, all distance measures data reported in our results (Figures 4–8) suggest that community variance in terms of member composition was most influenced by oral infection. This outcome is supported by the alpha indexes, which showed that the greatest diversity in terms of abundance and community evenness was observed in the PG-inoculated group prior to breeding and that the population became more even during pregnancy by GD 18.

When Bray-Curtis beta diversity analysis of consensus data included the sham-inoculated group, this point was positioned furthest from the center and was most distant from the baseline control point relative to PC1 (Figure 8), suggesting that the core consensus communities were most similar between groups sampled from the same animal set. In contrast, Euclidean beta diversity analysis showed that PG-inoculated group was the most dissimilar among the four groups along PC1 and PC3 axes, while the consensus points of the other three groups clustered together. Along PC2, however, the Sham-inoculated point was more dissimilar, particularly at the species level. Reported comparisons of different beta-diversity metrics used to compare community data (Legendre and de Caceres, 2013) suggest that Euclidean matrix is not appropriate when visualizing dissimilarity between individually plotted communities derived from complex samples (e.g., microbiomes from animals) since absence-presence data (without abundance) may put too high or low a value on each community member identified within each individual sample; however, it is useful to visualize dissimilarities between collective consensus community group data. It is important to recall that all of the animals used in this study came from the same colony and were housed the same way.

These data suggest that health and *P. gingivalis* inoculation induced periodontal disease guides microbial composition at the species level, that species level shifts are often reflective of their condition, and that species level dissimilarities typically become more pronounced during disease, irrespective of whether the samples were collected from the same animals (intra-group) or from a different set of animals. These data also support the concept that there are common core site-specific microbiomes among host species and that established microbiomes within an individual animal are resistant to gross dysbiotic shifts in response to host conditions when reviewed at higher taxonomic levels.

### Analyzing Baseline vs. Sham-Inoculated Groups Suggest a Less Diverse but Recovering Core Microbiome 12-Weeks Post-antibiotic Treatment

The standard protocol for inducing periodontitis in rats is to first prophylactically treat animals with antibiotics, then allow for a short microbial recovery time before starting inoculations (Kesavalu et al., 2007; Verma et al., 2010; Phillips et al., 2018). Prophylactic antibiotics are known to cause a chemically induced dysbiosis (Rogers et al., 2010; Manrique et al., 2013). Hence, we included pre-antimicrobial treatment samples in our study to potentially identify such perturbations. Despite there being overall similarities in the oral communities of baseline and sham-inoculated groups in terms of alpha diversity, we identified a larger population proportion of *Staphylococcaceae* and *Lactobacillaceae* in both the baseline and PG-inoculated group animals relative to the non-pregnant sham group animals, suggesting intra-animal microbiome stability and inter-animal differences would make it difficult to perform meaningful inter-animal group profile comparisons. Consequently, we primarily focused on intra-animal time course microbiome comparisons in this report. However, we did draw some basic conclusions from our inter-animal comparisons as follows.

Baseline was used as our pre-antibiotic, uninfected group while sham-inoculated was our time-matched vehicle control of an oral community recovering from antibiotic induced dysbiosis since it had 12 weeks of recovery time without addition of further stressors. As shown in Figure S1, *Pasteurellaceae* populations were similar between our control groups but the *Staphylococcaceae* family shifted from 43.56% at baseline to 55.4% at PG-inoculated but was only at 17.83% in the sham-inoculated group. Moreover, there was altered *Streptococcus* species diversity in the sham-inoculated group. As stated in our results, our alpha diversity findings as measured by Shannon and Simpson indices for overall community species richness (abundance and evenness) indicate that sham-inoculated and baseline were more similar to each other than to PG-inoculated group consensus profiles. This result agrees with the findings of Manrique et al. which indicated that while antibiotics reduce the overall abundance of bacteria, the core microbial community structure remains the same when given time to recover from the perturbation (Manrique et al., 2013). Overall, our data suggests that the oral flora in the sham-inoculated group, while not the same as baseline group was likely recovering its “normal” microbiome core profile characteristics by the end of the 12-week sham inoculation period.

### Infection and Pregnancy Produced an Altered Community Metabolic Profile Unique to Each Condition

When alpha and beta diversity analysis of the collective consensus microbiome of each group were compared (Figures 3, 8), we observed a shift in response to *P. gingivalis* inoculation that induced periodontal disease that seemed to then revert toward baseline during pregnancy. A broad survey of population proportions among a wide variety of identified families, including the top 5 most dominant families, supported this trend. Thus overall, this data suggests that the rat oral microbiome has a family level commonality at its core. However, between animal variability, particularly at lower taxonomic levels, indicates that the oral microbiome at pregnancy was actually not that similar to baseline, making comparative analyses challenging.

Notable trends, microbiome shifts, and predicted associations were discovered that suggest certain broad characteristics (e.g., Gram classification, cell morphology) traditionally used to describe oral microbial populations “typical” of health or disease were relatively inadequate indicators overall of dysbiosis when compared to our assessment of the metagenomic changes in the population in context of predicted metabolic shifts. For example, the oral community progressively became more populated with Gram-positive rods and less populated with Gram-negative cocci. In contrast, Gram-positive cocci and Gram-negative rod population proportions shifted in response to infection then shifted back in response to pregnancy. Altered microbiome diversity and dysbiosis was still evident in the pregnant group when assessed in context of oxygen requirements and predicted major metabolite production, the latter of which is covered in more detail in the next section. Although the population proportion of the obligate anaerobe *P. gingivalis* was larger in the pregnancy group, we observed that by GD 18 that the now orally infected and pregnant rats had a more aerobic oral community overall (Figure 10). We also identified oral colonization of previously undetected opportunistic and commensal bacteria (Supplementary Material). Considering both pregnancy and periodontal disease impact the inflammatory and immune response (Guncu et al., 2005; Wu et al., 2015), these conditions would likely result in the host being much more vulnerable to colonization by these previously undetected organisms.

### Insights and Limitations of the Predicted Morphological Classification, Oxygen Requirement, and Major Metabolite Production Profiles of Baseline, Infected, and Pregnant Animals

We found that the predicted population proportion of both lactic acid producers and propionate producers progressively decreased while butyrate producers progressively increased over time in response to *P. gingivalis* inoculation then in response to pregnancy by GD 18. Lactic acid production is generally associated with carbohydrate fermentation by oral *Streptococci* and other genera of the oral microbiome (McLean et al., 2012) which, combined with diet, contributes to excessive biofilm formation, low pH, and caries development in humans. When lactic acid production is reviewed in context of periodontal disease, our findings agree with consensus observations reported in the literature. Specifically, in a normal oral cavity during health, oral *Streptococci* typically dominate the oral microbial population but lose their foothold to periodontal pathogens, especially Gram-negative anaerobes (Takahashi, 2015).

Acetate, propionate, and butyrate are all short chain fatty acids (SCFAs), are produced by many bacteria by a variety of mechanisms, and have complex contextual associations with both health and disease. For example, propionate is a weaker acid than lactic acid, often generated though conversion of lactic acid, and is typically produced in the oral cavity by genera such as *Veillonella* and *Lactobacillus* (found in all of our groups), and *Clostridium* (absent in our control groups). Lactic acid conversion to propionate contributes to acid neutralization, and is believed to facilitate dominance of the more acid sensitive *Streptococci* species associated with dental health as well as facilitating growth of acid sensitive periodontal pathogens such as *Porphyromonas* (Takahashi, 2015). Both butyrate and propionate are major metabolites produced by oral bacteria such as *Porphyromonas* and *Clostridium* that use proteins and amino acids as a primary carbon source, typically co-generating ammonia though amino acid deamination, further contributing to acid neutralization (Takahashi, 2015). SCFA production is generally associated with health when produced in the gut by colonic bacteria, typically through fermentation of dietary fiber. Specific metabolites such as butyrate, when provided in a dietary supplement, have shown beneficial health effects in the host (van Immerseel et al., 2010; Herrema et al., 2017). In contrast, butyrate production in the oral cavity is reported to be cytotoxic in patients with oral disease, such as periodontal infection, and has been shown to be responsible for the release of reactive oxygen species in chronic periodontitis (Anand et al., 2016). These reported associations highlight the importance of site-specific studies in context of host-pathogen interactions. When delving into the particulars of microbial butyrate production among pathogens and commensals, Anand et al. showed that these organisms have evolved distinct pathways where, unlike typical commensal species, species that are recognized as pathogens typically co-generate harmful byproducts like ammonia along with butyrate, which explains how butyrate production could be associated with both health and disease (Anand et al., 2016).

The microbial metabolite 2, 3-butanediol is typically produced during carbohydrate fermentation. Major production of this metabolite by certain commensal oral *Streptococci*, when in association with the opportunistic environmental pathogen *Pseudomonas aeruginosa* in the lungs of cystic fibrosis patients, is associated with chronic disease and it is generally considered to be cytotoxic to human cells at high concentrations and long term exposure (Whiteson et al., 2017). Interestingly, *in vitro* work with dendritic cells showed that 2, 3-butanediol has an anti-inflammatory affect when co-incubated at below toxic concentrations, but it was suggested that inhibition of the immune response in tissues such as the mucosa may ultimately be detrimental, contributing to chronic infection (Whiteson et al., 2017). There is no known health benefit of 2, 3-butanediol in humans, whether it is synthesized by bacteria or formed in mammalian cells, especially in the liver after ethanol consumption. Our data showed that the predicted population proportion of 2, 3-butanediol producers relative to baseline increased in response to oral PG-inoculation then decreased during pregnancy by GD 18. This outcome is consistent with having a dysbiotic or “unhealthy” microbiome post PG-inoculation.

Riboflavin (Vitamin B2) is an essential precursor used to form the major coenzymes flavin mononucleotide (FMN) and flavin adenine dinucleotide (FAD), leading to the synthesis of vitamin B3 and B6 respectively, as well as other forms of Vitamin B and numerous flavoproteins. Riboflavin is synthesized by a wide variety of bacteria. Some bacterial species (e.g., most Firmicutes and Bacteroidetes) are high producers of riboflavin, secreting excess into their environment. Because riboflavin is essential for growth of all living cells, some bacterial species import riboflavin synthesized by other bacteria because they are either poor or non-producers. Humans cannot synthesize or store these water-soluble B vitamins and are dependent on diet and on biosynthesis by resident bacteria (Magnusdottir et al., 2015). Because bacterial non-producers compete with host cells for available riboflavin, understanding the mechanisms the host uses to preserve health in the face of microbial mechanisms used to survive in the host, in context of microbially produced essential metabolites like riboflavin, may provide insights into the etiology of observed microbiome shifts. For example, bacterially produced riboflavin is believed to play a direct role in immune function in the host though at least two mechanisms. Riboflavin is associated with reactive oxygen species generation in innate immune cells through priming of NADPH oxidase (Yoshii et al., 2019). Intermediates formed during microbial riboflavin biosynthesis also activate MAIT (mucosal-associated invariant T) cells through binding to the MR1 protein of MHC-I molecules on antigen presenting cells (Eckle et al., 2015; Yoshii et al., 2019). However, stimulation by commensal microorganisms are insufficient to fully elicit MAIT cell effector function (Berkson and Prlic, 2017). Thus, commensal bacterial mediated priming of MAIT cells is proposed to contribute to their immunological role in pathogen surveillance, which are then fully activated in the presence of pathogens at sufficient microbial load. Consequently, the presence of riboflavin biosynthesis intermediates has reportedly been used as a biomarker for microbial infection (Eckle et al., 2015).

Although we inoculated the oral cavity of rats with *P. gingivalis*, a robust and self-sufficient riboflavin producer, the consensus microbiome post-inoculation in animals with periodontal disease did not reflect overall increased community riboflavin production. Rather, we observed that the oral microbial population's predicted ability to synthesize riboflavin decreased by ~50% in response to infection and then rebounded to surpass baseline levels during pregnancy by GD 18. With shallow understanding, this outcome would seem to contradict an expectation of high riboflavin production due to infection by *P. gingivalis*. However, presuming riboflavin production is generally beneficial in mammalian mucosa as reported in the literature for the GI tract, our data would be consistent with development of an “unhealthy” microbiome followed by recovery to a more beneficial microbial profile when viewed in terms of riboflavin production. This highlights how the consensus microbiome profile and its metabolome may better reflect important aspects of microbial health or dysbiosis that would not be revealed if only targeted characterization of a specific metabolic contribution of individual pathogens identified within a community was performed. Yet in this context, knowing the site-specific host response to individual pathogens increases our overall understanding of the disease process.

It is worth noting that the levels of MAIT cells found in different tissues sites differ, presumably impacting the local inflammatory response outcomes. Specifically, the abundance of MAIT cells in human apical periodontal tissues are reported to be similar to levels found in peripheral blood but markedly higher than levels found in gingival tissues (Davanian et al., 2019) and lower than levels found in the walls of the large intestine (epithelium and lamina propria) (Hama et al., 2019). Overall, microbial species that act as pathogens at sufficient microbial load, such as *P. gingivalis*, and that are also efficient riboflavin producers would presumably contribute to the localized chronic inflammatory response characteristic of periodontal disease. However, riboflavin producing pathogens may induce a more robust MAIT cell response in the intestines compared to the gingiva, or perhaps have a lower pathogen load threshold, due to the higher levels of resident MAIT cells. How increased or decreased riboflavin availability may further modulate the microbiome profile or disease development due to inherent differences in the environment, microbial load, and site-specific tissue characteristics of the gingiva are unknown and should be further investigated.

Collectively, our outcomes and current understanding shined a light on certain metabolic characteristics in the context of the observed microbiome profiles that we believe should be the target of future studies, including directly assessing select metabolic shifts of the oral microbial population in context of oral infection and pregnancy. However, we recognize that there are several limitations to this pilot study that may have negatively impacted our interpretations. A key limitation in interpreting microbiome data in general lies in the dependence on reference databases to create each microbiome profile. Because unmapped OTUs (Figure S3) are essentially discarded, metagenomic analyses can only “discover” and infer characteristics about the microbial community relative to what is already “known.” In the future, when more reference genomes become available, these data can be reanalyzed to map the un-mapped reads. Similarly, in this study, we observed a large number of slash calls, thus limiting the level of the taxonomic hierarchy we could confidently compare between microbiomes. This is likely due to similarities of the particular 16S variable region sequence between closely related species. However, it is possible that with additional reference genomes, these slash calls might be differentiated.

When we designed this project, we opted to focus our pilot study on determining the longitudinal intra-animal shifts in the oral microbiome in response to *P. gingivalis* inoculation and pregnancy and did not assess the oral microbiome of sham-inoculated pregnant rats. It would be interesting to parse out the impact of pregnancy alone on the oral community in a future study.

## Data Availability Statement

The raw data supporting the conclusions of this article will be made available by the authors, without undue reservation, to any qualified researcher.

## Ethics Statement

The animal study was reviewed and approved by University of Wisconsin Animal Care and Use Committee, University of Wisconsin–Madison.

## Author Contributions

PP and her graduate student MW conducted all sample processing, sequencing, and sequencing data analysis. LR and her lab technician GP conducted all animal experiments, animal pathology assessments, and sample collection. PP, LR, AP-F, and MB are coinvestigators that together planned, developed, and sought funding for several collaborative projects that specifically targeted the role of oral pathogens in disease development in context of pregnancy. PP, LR, and MW are the primary writers of the manuscript with editorial contributions by the rest of the co-authors.

### Conflict of Interest

The authors declare that the research was conducted in the absence of any commercial or financial relationships that could be construed as a potential conflict of interest.
