# Supplementary material for: A Uniquely Altered Oral Microbiome Composition Was Observed in Pregnant Rats With Porphyromonas gingivalis Induced Periodontal Disease
Source: Front Cell Infect Microbiol. 2020 Mar 6;10:92. doi: 10.3389/fcimb.2020.00092 (PMC7069352; doi:10.3389/fcimb.2020.00092)
Supplement: Supplementary file 1 [file Presentation_1.pptx]

## Slide 1
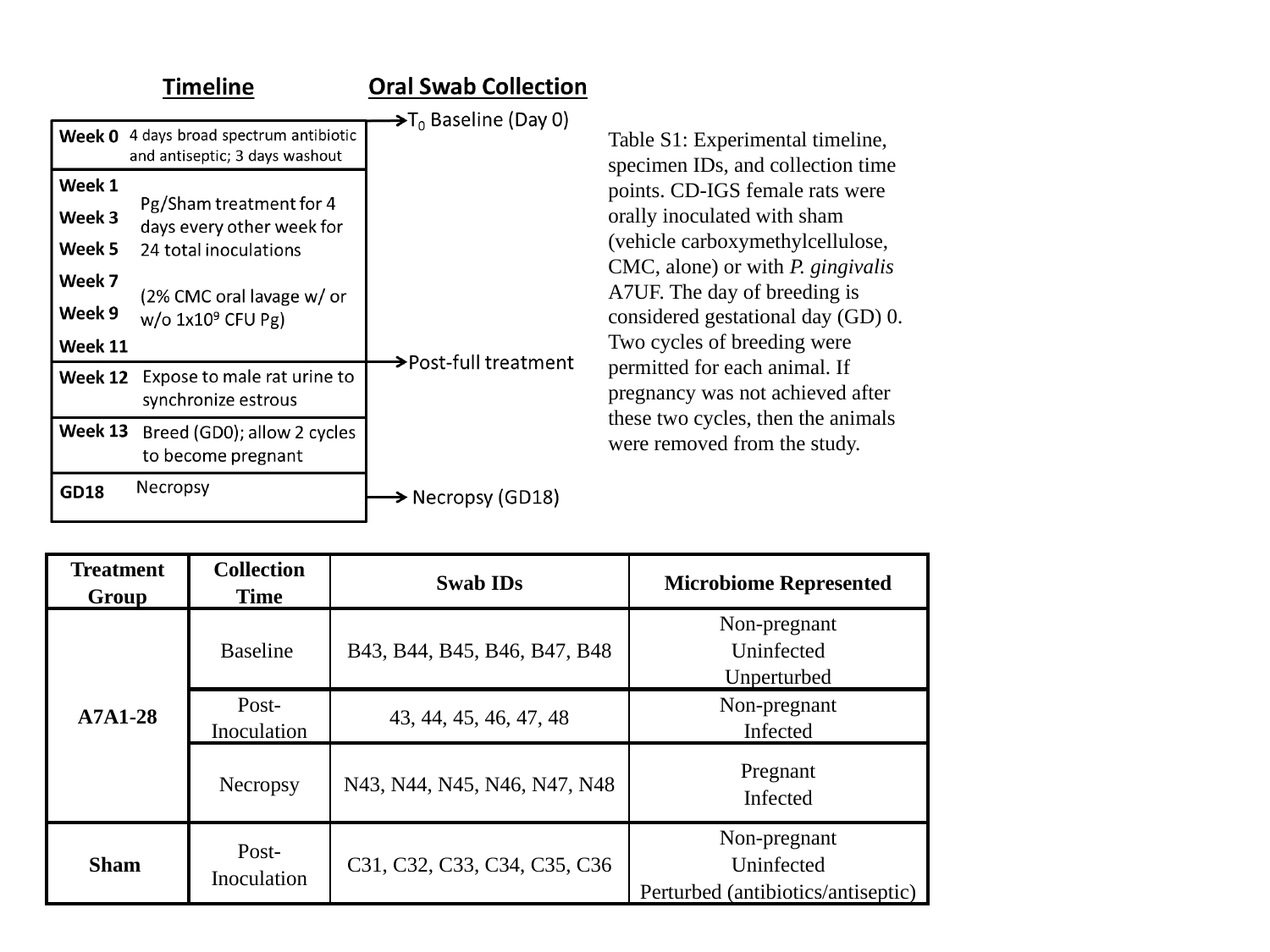

Table S1: Experimental timeline, specimen IDs, and collection time points. CD-IGS female rats were orally inoculated with sham (vehicle carboxymethylcellulose, CMC, alone) or with P. gingivalis A7UF. The day of breeding is considered gestational day (GD) 0. Two cycles of breeding were permitted for each animal. If pregnancy was not achieved after these two cycles, then the animals were removed from the study.
| Treatment Group | Collection Time | Swab IDs | Microbiome Represented |
| --- | --- | --- | --- |
| A7A1-28 | Baseline | B43, B44, B45, B46, B47, B48 | Non-pregnant Uninfected Unperturbed |
| | Post-Inoculation | 43, 44, 45, 46, 47, 48 | Non-pregnant Infected |
| | Necropsy | N43, N44, N45, N46, N47, N48 | Pregnant Infected |
| Sham | Post-Inoculation | C31, C32, C33, C34, C35, C36 | Non-pregnant Uninfected Perturbed (antibiotics/antiseptic) |

## Slide 2
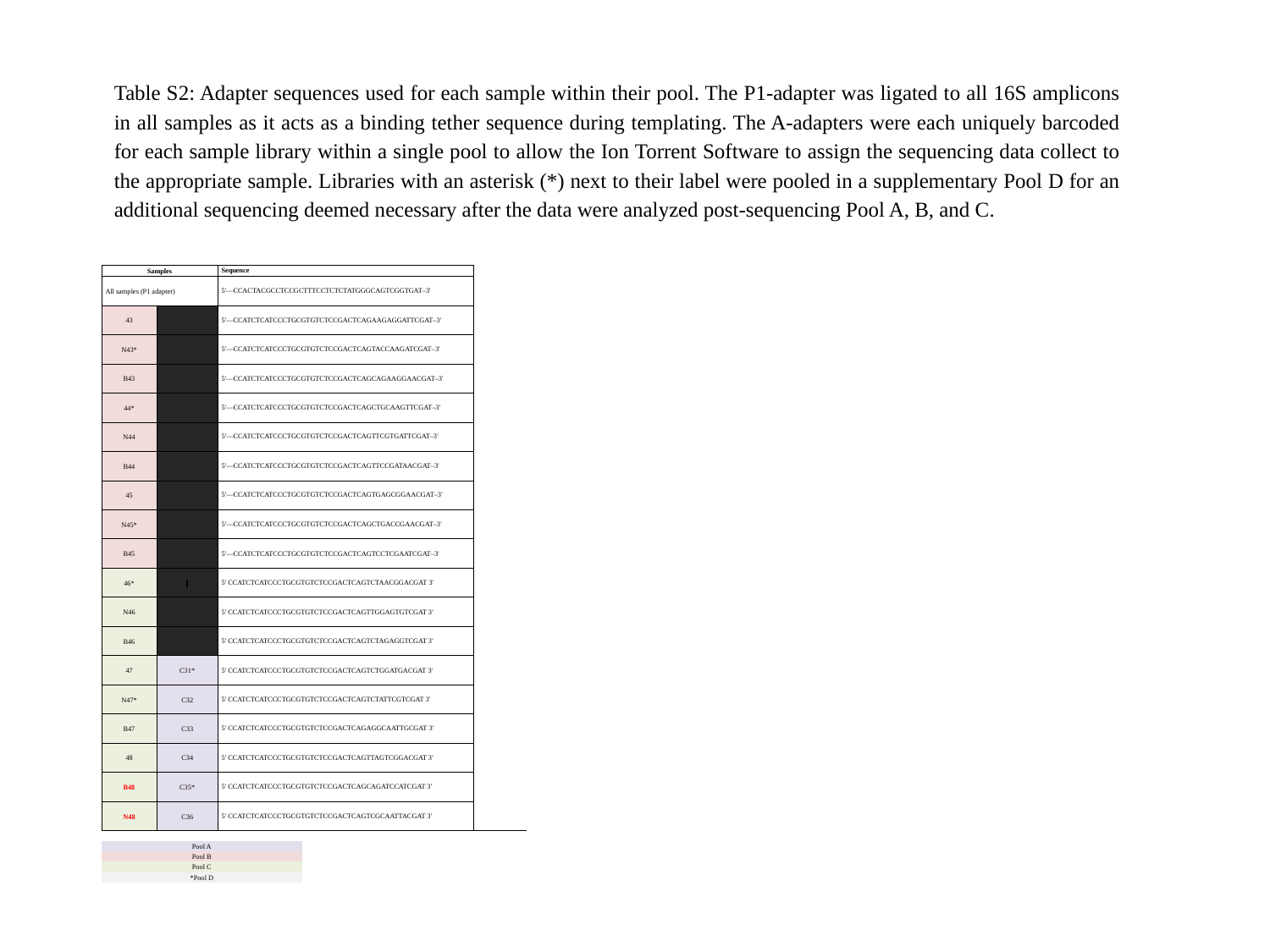

Table S2: Adapter sequences used for each sample within their pool. The P1-adapter was ligated to all 16S amplicons in all samples as it acts as a binding tether sequence during templating. The A-adapters were each uniquely barcoded for each sample library within a single pool to allow the Ion Torrent Software to assign the sequencing data collect to the appropriate sample. Libraries with an asterisk (*) next to their label were pooled in a supplementary Pool D for an additional sequencing deemed necessary after the data were analyzed post-sequencing Pool A, B, and C.
| Samples | | Sequence | | | | |
| --- | --- | --- | --- | --- | --- | --- |
| All samples (P1 adapter) | | 5'—CCACTACGCCTCCGCTTTCCTCTCTATGGGCAGTCGGTGAT–3' | | | | |
| 43 | | 5'—CCATCTCATCCCTGCGTGTCTCCGACTCAGAAGAGGATTCGAT–3' | | | | |
| N43\* | | 5'—CCATCTCATCCCTGCGTGTCTCCGACTCAGTACCAAGATCGAT–3' | | | | |
| B43 | | 5'—CCATCTCATCCCTGCGTGTCTCCGACTCAGCAGAAGGAACGAT–3' | | | | |
| 44\* | | 5'—CCATCTCATCCCTGCGTGTCTCCGACTCAGCTGCAAGTTCGAT–3' | | | | |
| N44 | | 5'—CCATCTCATCCCTGCGTGTCTCCGACTCAGTTCGTGATTCGAT–3' | | | | |
| B44 | | 5'—CCATCTCATCCCTGCGTGTCTCCGACTCAGTTCCGATAACGAT–3' | | | | |
| 45 | | 5'—CCATCTCATCCCTGCGTGTCTCCGACTCAGTGAGCGGAACGAT–3' | | | | |
| N45\* | | 5'—CCATCTCATCCCTGCGTGTCTCCGACTCAGCTGACCGAACGAT–3' | | | | |
| B45 | | 5'—CCATCTCATCCCTGCGTGTCTCCGACTCAGTCCTCGAATCGAT–3' | | | | |
| 46\* | | 5' CCATCTCATCCCTGCGTGTCTCCGACTCAGTCTAACGGACGAT 3' | | | | |
| N46 | | 5' CCATCTCATCCCTGCGTGTCTCCGACTCAGTTGGAGTGTCGAT 3' | | | | |
| B46 | | 5' CCATCTCATCCCTGCGTGTCTCCGACTCAGTCTAGAGGTCGAT 3' | | | | |
| 47 | C31\* | 5' CCATCTCATCCCTGCGTGTCTCCGACTCAGTCTGGATGACGAT 3' | | | | |
| N47\* | C32 | 5' CCATCTCATCCCTGCGTGTCTCCGACTCAGTCTATTCGTCGAT 3' | | | | |
| B47 | C33 | 5' CCATCTCATCCCTGCGTGTCTCCGACTCAGAGGCAATTGCGAT 3' | | | | |
| 48 | C34 | 5' CCATCTCATCCCTGCGTGTCTCCGACTCAGTTAGTCGGACGAT 3' | | | | |
| B48 | C35\* | 5' CCATCTCATCCCTGCGTGTCTCCGACTCAGCAGATCCATCGAT 3' | | | | |
| N48 | C36 | 5' CCATCTCATCCCTGCGTGTCTCCGACTCAGTCGCAATTACGAT 3' | | | | |
| | | | | | | |
| Pool A | | | | | | |
| Pool B | | | | | | |
| Pool C | | | | | | |
| \*Pool D | | | | | | |
| | | | | | | |

## Slide 3
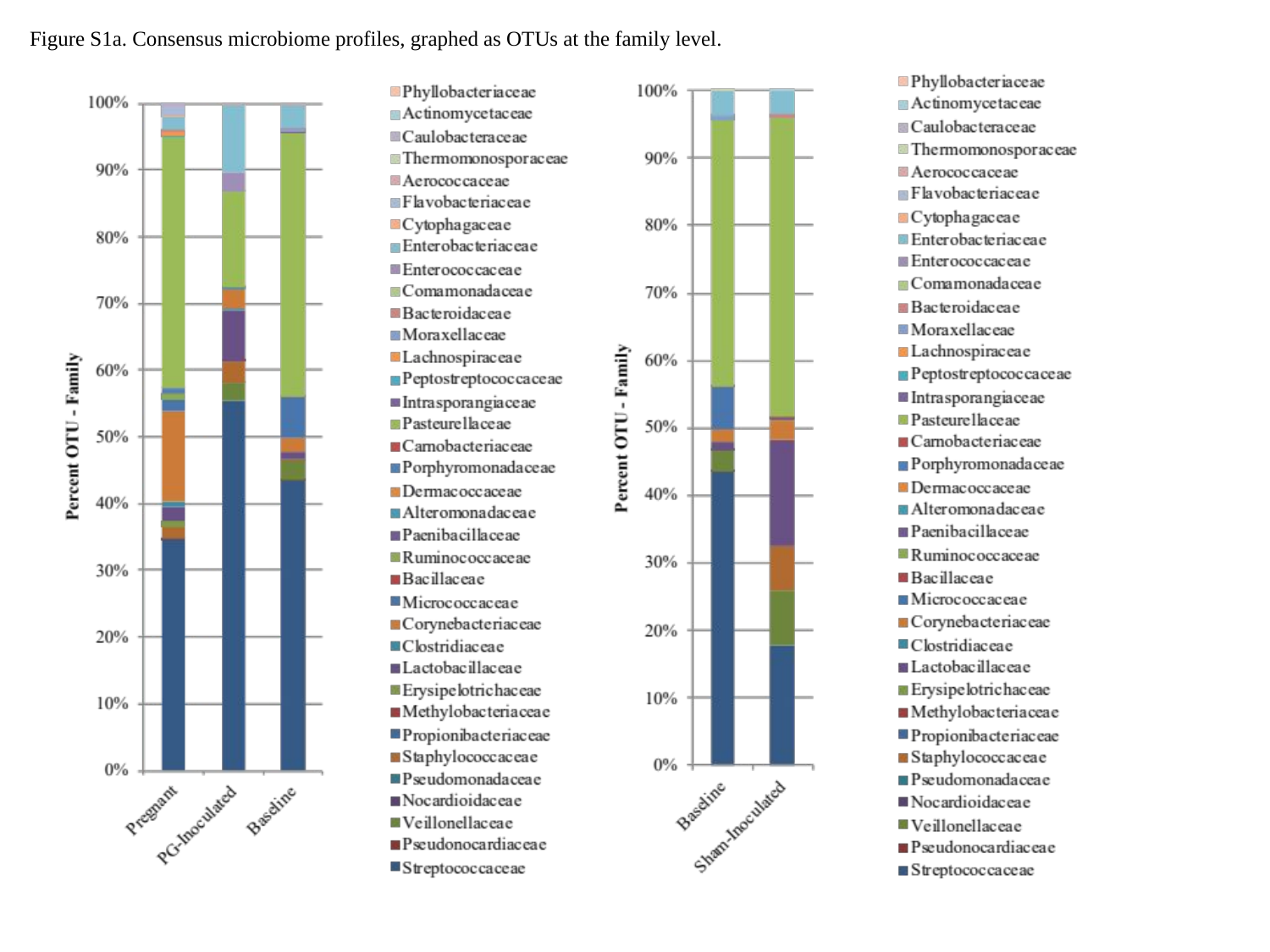

Figure S1a. Consensus microbiome profiles, graphed as OTUs at the family level.

## Slide 4
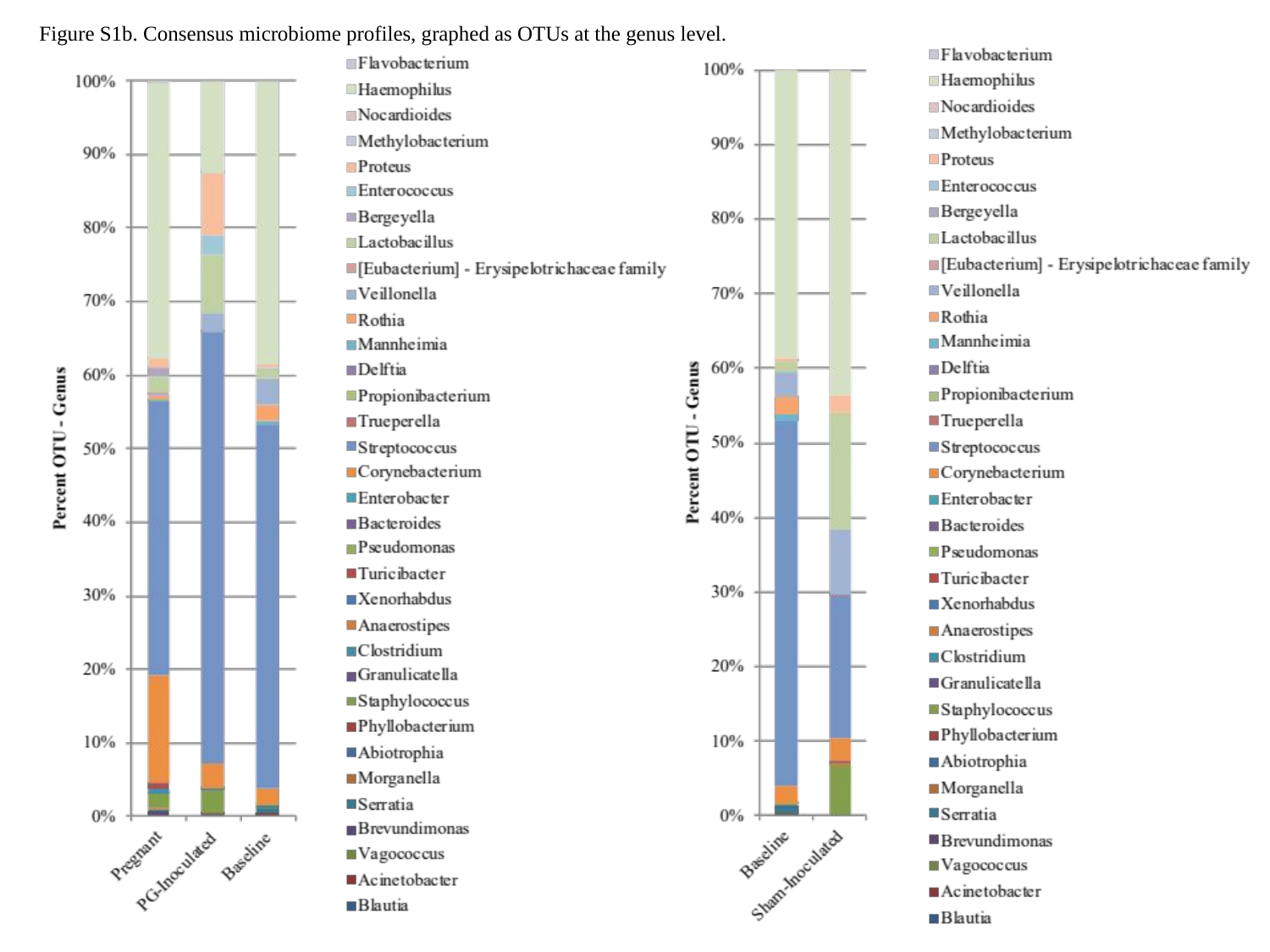

Figure S1b. Consensus microbiome profiles, graphed as OTUs at the genus level.

## Slide 5
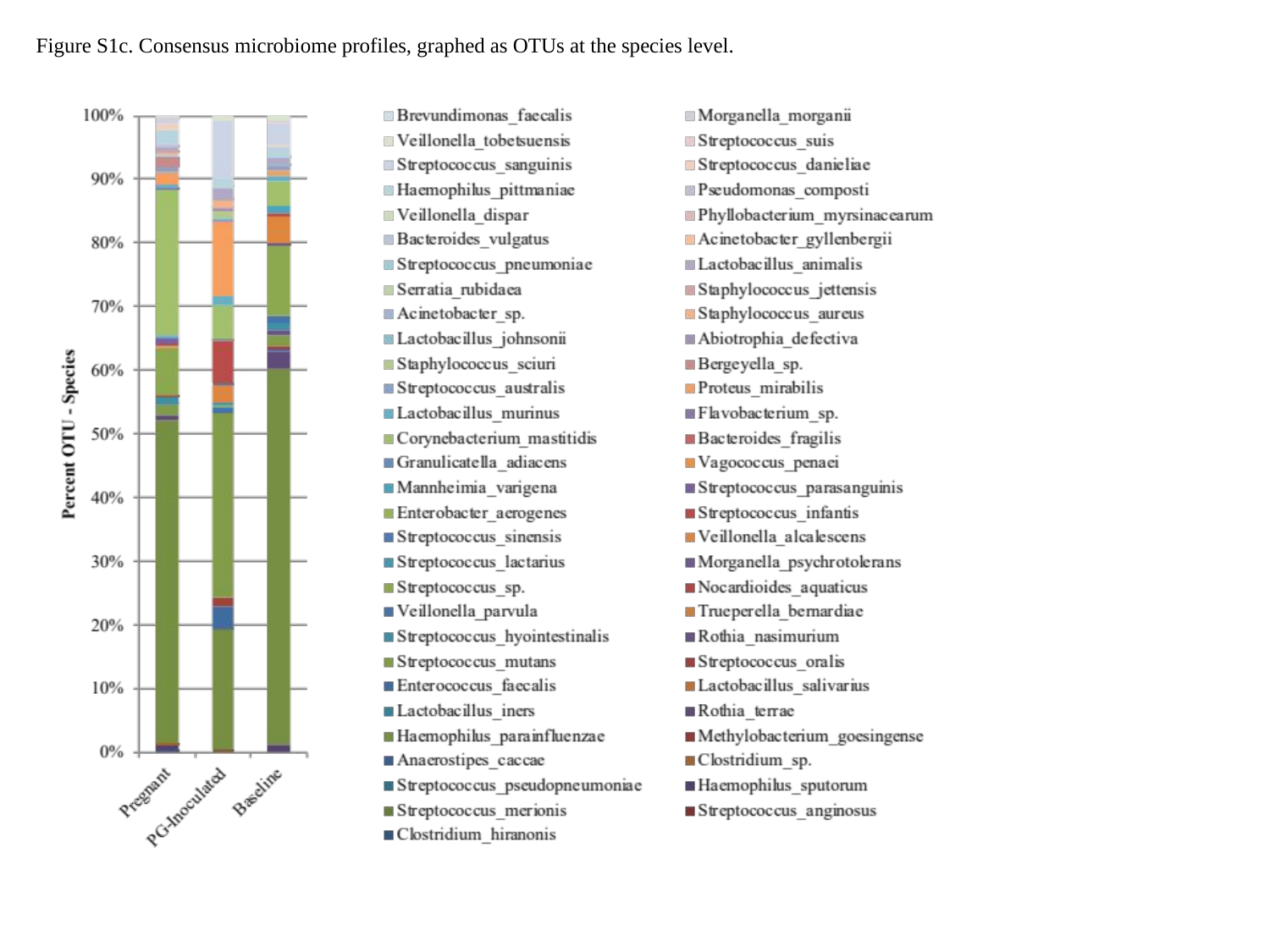

Figure S1c. Consensus microbiome profiles, graphed as OTUs at the species level.

## Slide 6
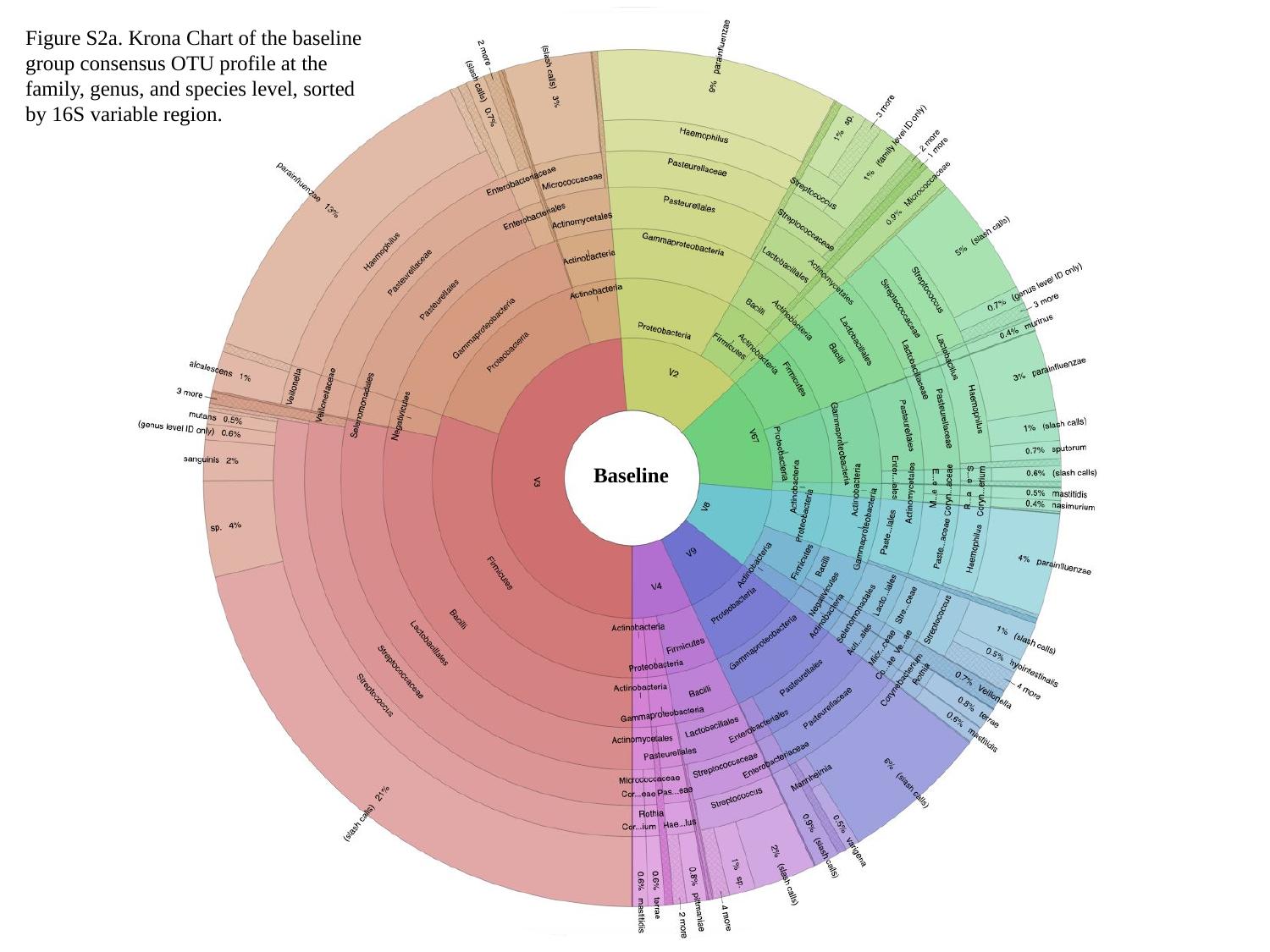

Baseline
Figure S2a. Krona Chart of the baseline group consensus OTU profile at the family, genus, and species level, sorted by 16S variable region.

## Slide 7
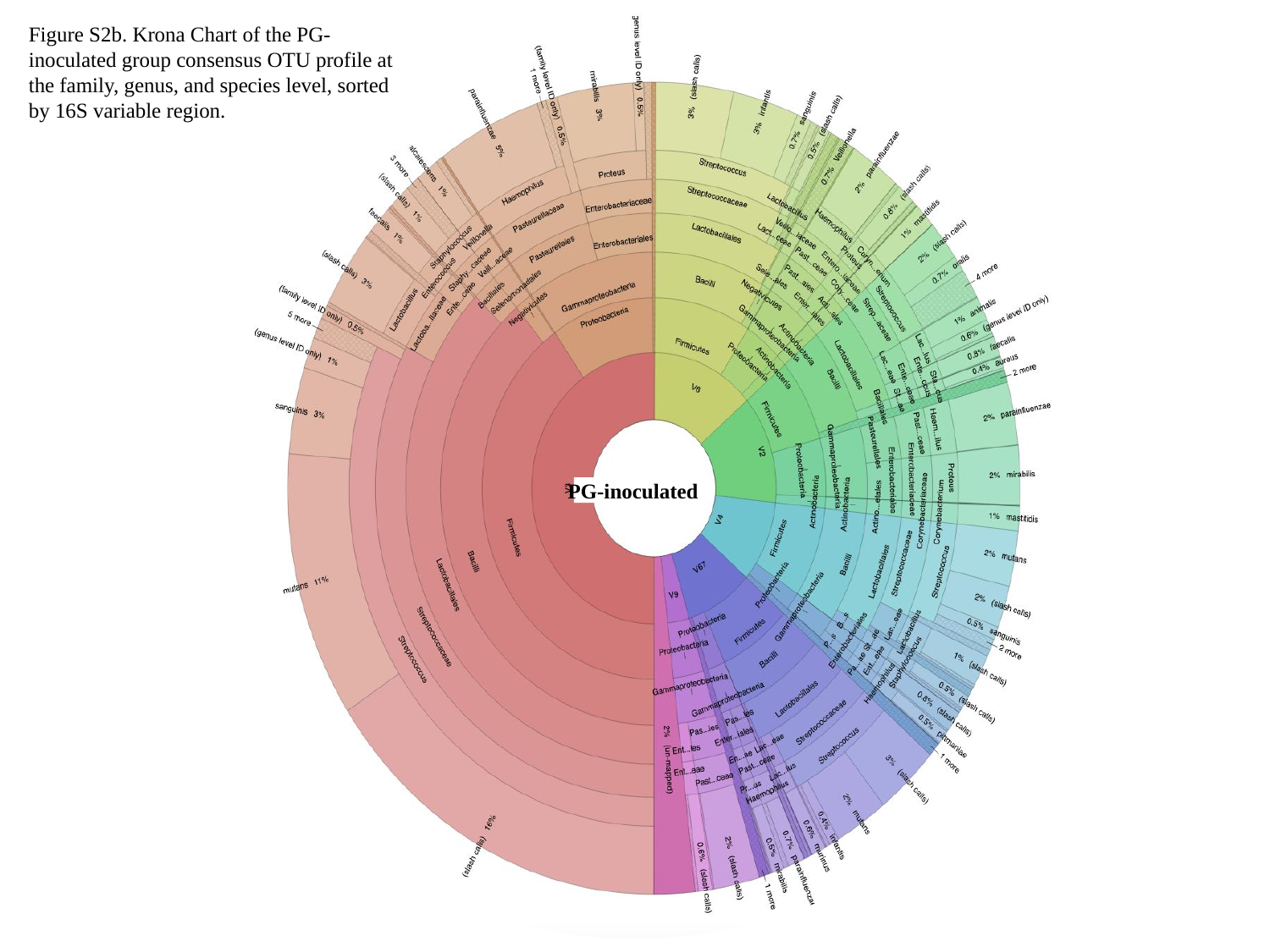

PG-inoculated
Figure S2b. Krona Chart of the PG-inoculated group consensus OTU profile at the family, genus, and species level, sorted by 16S variable region.

## Slide 8
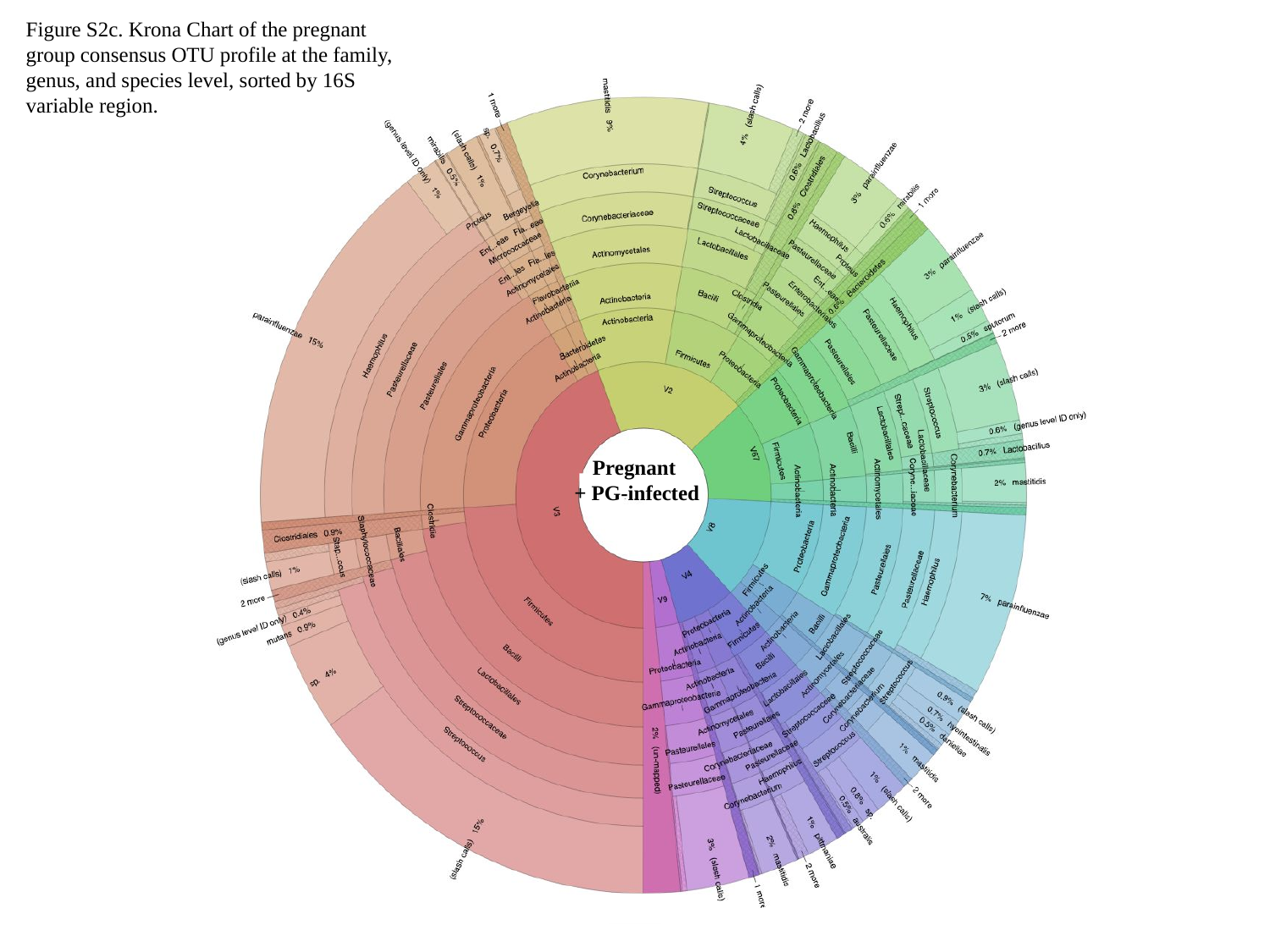

Figure S2c. Krona Chart of the pregnant group consensus OTU profile at the family, genus, and species level, sorted by 16S variable region.
Pregnant
+ PG-infected

## Slide 9
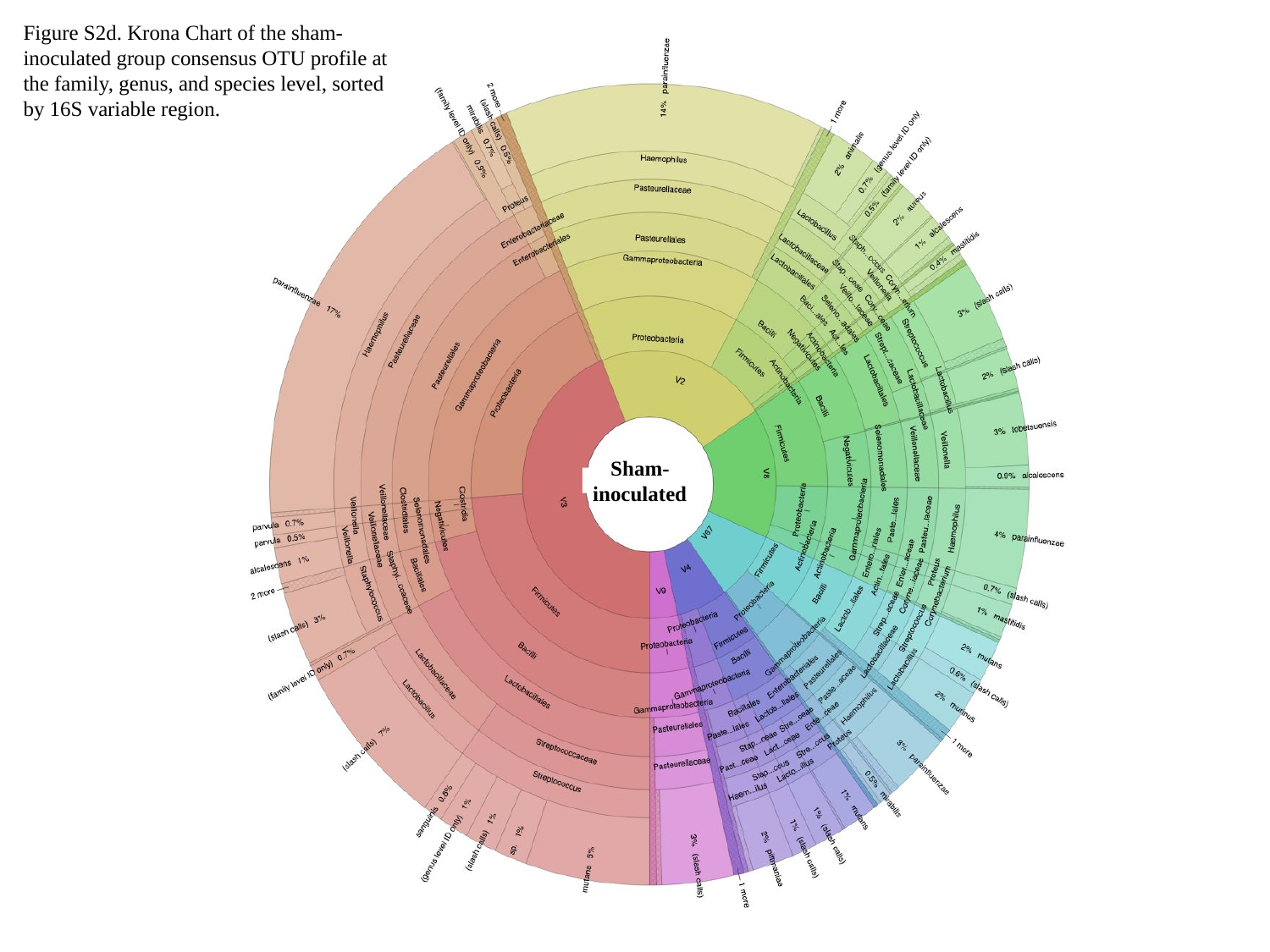

Figure S2d. Krona Chart of the sham-inoculated group consensus OTU profile at the family, genus, and species level, sorted by 16S variable region.
Sham-
inoculated

## Slide 10
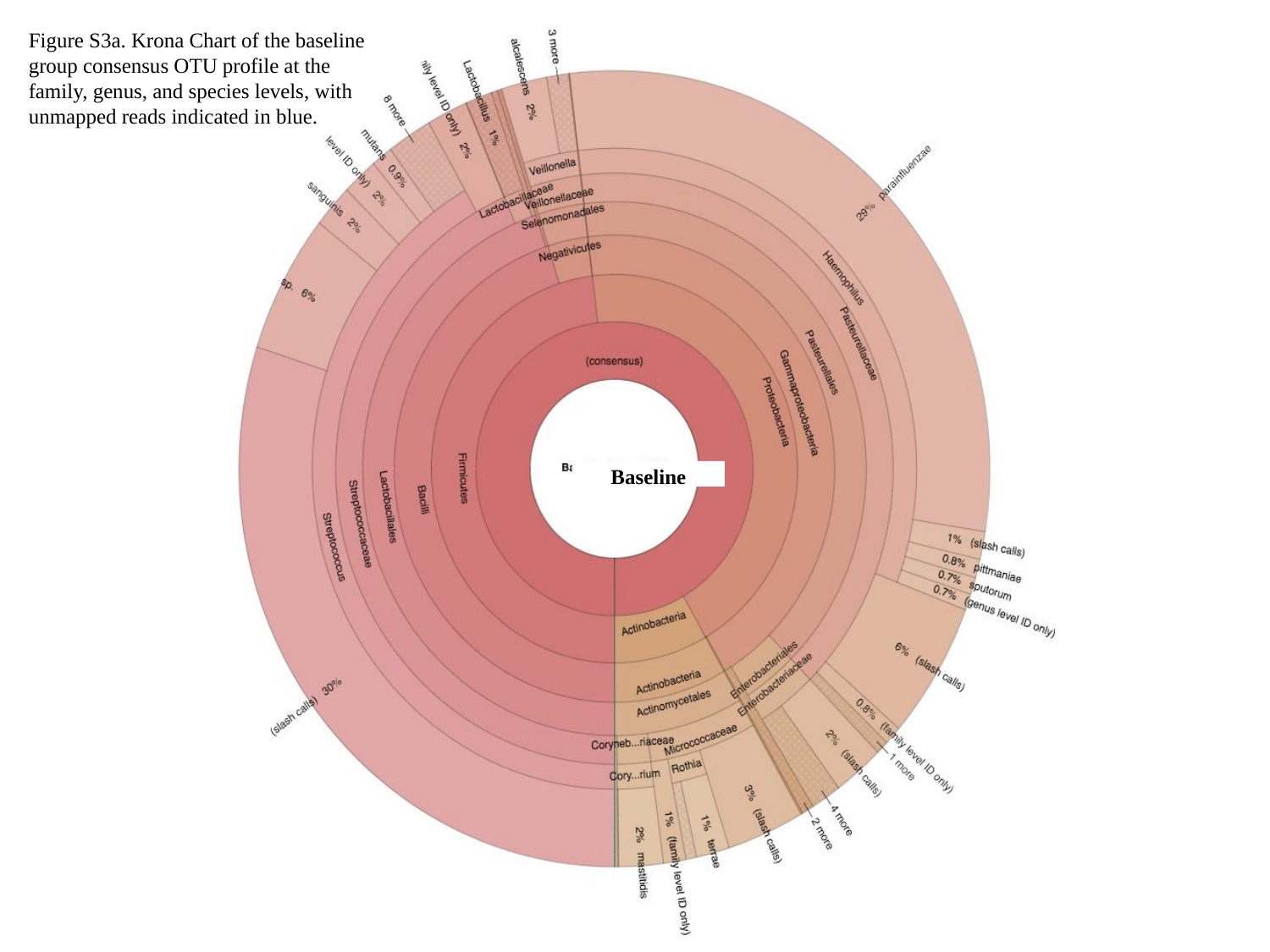

Figure S3a. Krona Chart of the baseline group consensus OTU profile at the family, genus, and species levels, with unmapped reads indicated in blue.
Baseline

## Slide 11
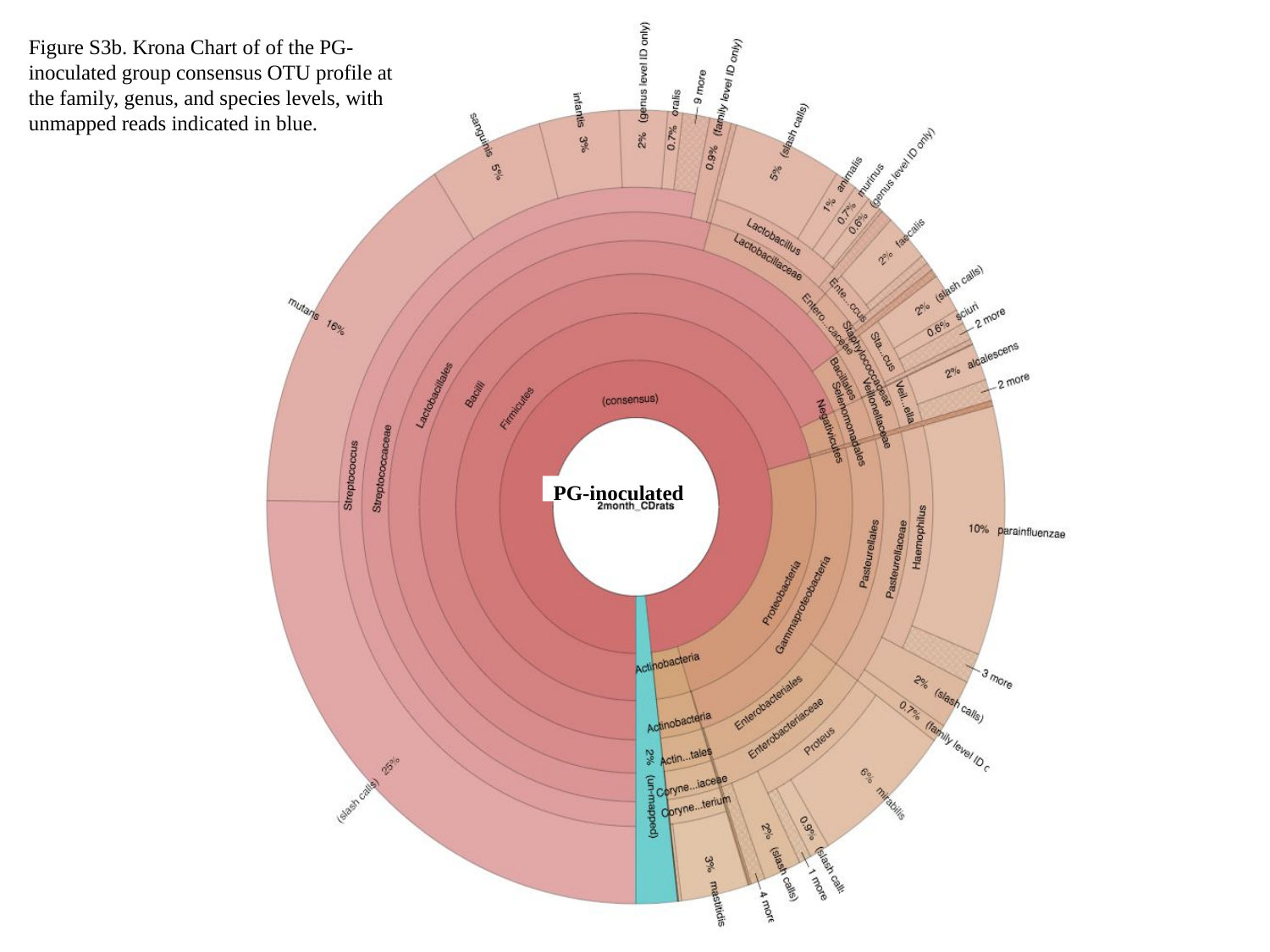

PG-inoculated
Figure S3b. Krona Chart of of the PG-inoculated group consensus OTU profile at the family, genus, and species levels, with unmapped reads indicated in blue.

## Slide 12
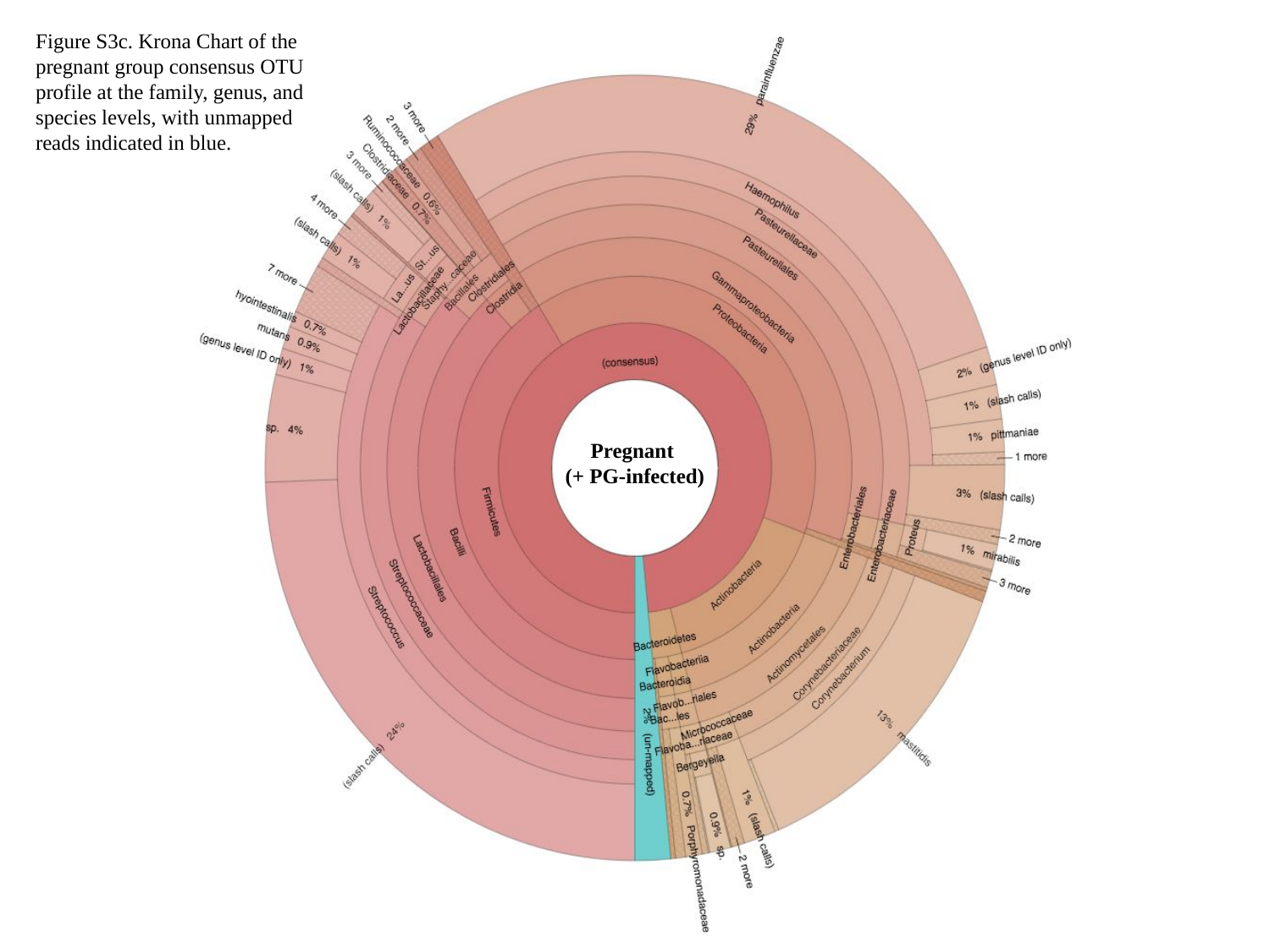

Pregnant
(+ PG-infected)
Figure S3c. Krona Chart of the pregnant group consensus OTU profile at the family, genus, and species levels, with unmapped reads indicated in blue.

## Slide 13
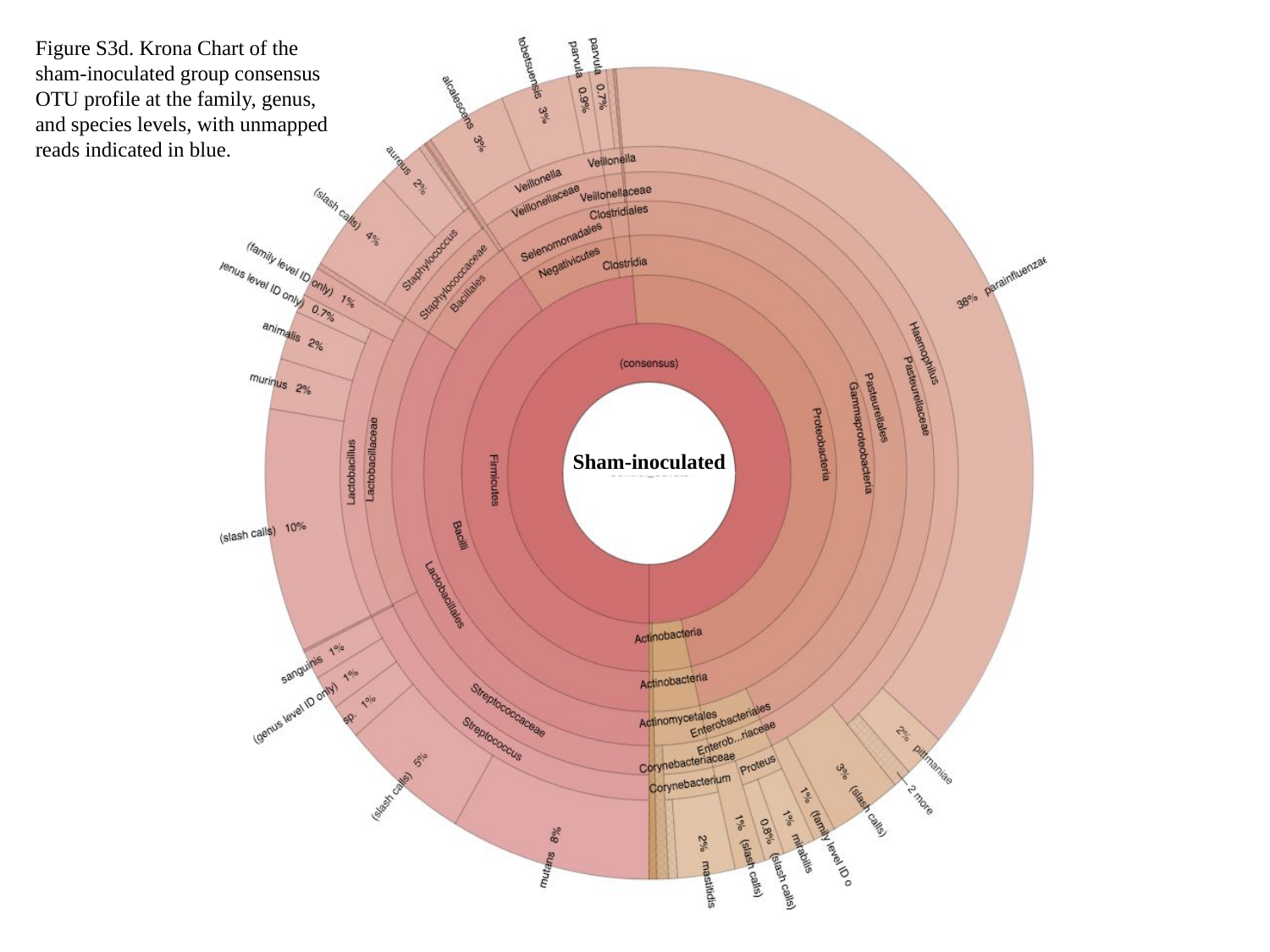

Sham-inoculated
Figure S3d. Krona Chart of the sham-inoculated group consensus OTU profile at the family, genus, and species levels, with unmapped reads indicated in blue.
